# A Method for Estimating Resource Use and Costs when Empirical Data Are Unavailable: Expert Elicitation Study Using the Example of Melanoma

**DOI:** 10.1177/23814683261451278

**Published:** 2026-06-25

**Authors:** Rob Hainsworth, Louisa Collins, Martin Eden, Adele Green, Paul Lorigan, Gabriel Rogers, Amber Salisbury, Katherine Payne

**Affiliations:** Division of Population Health, Health Services Research and Primary Care, Manchester Centre for Health Economics, School of Health Sciences, The University of Manchester, Manchester, UK; Population Health Department, QIMR Berghofer Medical Research Institute, Brisbane, QLD, Australia; School of Nursing, Queensland University of Technology (QUT), Brisbane, QLD, Australia; School of Public Health, University of Queensland, Brisbane, QLD, Australia; Division of Population Health, Health Services Research and Primary Care, Manchester Centre for Health Economics, School of Health Sciences, The University of Manchester, Manchester, UK; Population Health Department, QIMR Berghofer Medical Research Institute, Brisbane, QLD, Australia; Cancer Research UK Manchester Institute, The University of Manchester, Manchester, UK; The Christie NHS Foundation Trust, Manchester, UK; Division of Cancer Sciences, School of Medical Sciences, The University of Manchester, Manchester, UK; Division of Population Health, Health Services Research and Primary Care, Manchester Centre for Health Economics, School of Health Sciences, The University of Manchester, Manchester, UK; Division of Population Health, Health Services Research and Primary Care, Manchester Centre for Health Economics, School of Health Sciences, The University of Manchester, Manchester, UK; Division of Population Health, Health Services Research and Primary Care, Manchester Centre for Health Economics, School of Health Sciences, The University of Manchester, Manchester, UK

**Keywords:** melanoma, costs, resource use, expert elicitation

## Abstract

**Purpose.** The diagnosis, management, and therapy of cancer are rapidly advancing and becoming more costly. Data linking the diagnosis, management, and prescription of systemic anticancer therapy (SACT) are not publicly available, are time-consuming to obtain, and are onerous to analyze. We aimed to illustrate how a simple expert elicitation method can be used rapidly to calculate the cost of diagnosing and treating a cancer by stage of disease. We illustrate the method using the example of melanoma. **Methods.** We designed a simple structured elicitation exercise for melanoma experts from the United Kingdom with the aim of describing the diagnosis, management, and SACT prescription for people with melanoma and the associated proportions offered each option. We modeled experts’ beliefs using scaled beta distributions. We used random-effects meta-analysis to combine the estimates. Published unit costs (£; 2024–2025) were multiplied by estimated proportions to calculate the mean costs for the diagnosis, management, and SACT by disease stage. **Results.** Seven dermatologists, 5 oncologists, and 4 surgeons participated (2022–2023). There was variation in the estimates of the proportions receiving possible diagnosis, management, or SACT options. Diagnosing suspicious lesions cost £424 to £699 depending on the investigations required. Mean (95% confidence intervals) management costs were £782 (£611, £981) for stage 0, £946 (£776, £1,145) for stage 1a, £4,729 (£4,532, £4,944) for stage 1b/2, £3,719 (£3,213, £4,150) for macroscopic 3, and £2,827 (£2,757, £2,904) for microscopic 3. Mean SACT costs were £65,289 (£50,581, £74,521) for stage 3 and £134,065 (£115,787, £152,938) for stage 4. **Conclusions.** Our new approach to eliciting and combining estimates balances practical and theoretical considerations. We illustrate how this method produces estimates of the costs, and associated uncertainty, of the diagnosis, management, and SACT for melanoma by stage of disease in the absence of readily accessible diagnosis, management, and prescribing data. These cost estimates were not validated against registry data.

**Highlights:**

- Linked diagnosis, primary surgical management (hereafter “management”), and prescribing of systemic anticancer therapies (SACT) data describing the number and proportion of people with cancer offered these options are not readily available. The time to obtain data together with the time to conduct onerous analyses are a key barrier to obtaining values for use by decision analysts building economic models for evaluating new options for the early detection or prevention of cancer.
- We designed a simple expert elicitation exercise, completed in Excel. The exercise was designed to provide estimates of the total cost of cancer by stage of disease and crucially also to understand the extent of uncertainty around these estimates. We used melanoma as an exemplar cancer.
- Clinical experts (consultant dermatologists, surgeons, and oncologists) estimated the proportions of people offered possible diagnostic, management, or SACT options for melanoma (by stage of disease). Our study suggested that they found the expert elicitation exercise easy to understand and managed to complete the task.
- Random-effects meta-analysis used to pool the individual estimates indicated substantial heterogeneity across experts’ estimates of most elicited proportions. This finding suggested wide variation in possible approaches to the diagnosis, management, and use of SACT for melanoma.

Strategies to prevent cancers or detect them early (“early detection”) are important public health measures with the potential to improve population health while using health care resources more effectively.^1,2^ Generating the economic evidence to decide whether to implement these prevention or early-detection strategies involves understanding their impact on subsequent diagnosis, management, and treatment options and associated costs. Melanoma is one type of cancer that can be effectively prevented by, for example, influencing people to avoid direct exposure to ultraviolet light by using sunscreen or avoiding the use of sunbeds.^3,4^ Melanoma requires multidisciplinary teams to offer appropriate diagnosis, primary surgical management (hereafter “management”), and systemic anticancer therapies (SACT). The 2021 Global Burden of Disease study found that melanoma was responsible for 0.9% of deaths and 0.6% of disability-adjusted life-years worldwide.^5^

Since 2018, when Wilson and colleagues^6^ last costed the management of melanoma in the United Kingdom, the number of options has expanded rapidly, resulting in a complex array of approaches for the diagnosis, management, and use of SACT. The types of SACT used to treat stage 3 and stage 4 melanoma are evolving especially quickly and tend to have substantially higher costs than previously available treatments do.^7^ National Institute for Health and Care Excellence (NICE) guidelines also recommend diagnostic tests at an earlier stage of disease, consequently increasing diagnostic costs.^8^

Generating costs for the diagnosis, management, and treatment of cancer using observational data is slow. It is also resource intensive in terms of researcher time and costly due to the onerous tasks associated with data cleaning and subsequent analysis. The delay is because datasets for each element (diagnosis, management, SACT) need to be linked together. The often-observed delay in obtaining the required linked dataset means that economic evaluations of cancer early detection and prevention strategies generally rely on out-of-date estimates. The required estimates being the proportion of people with melanoma offered specific diagnostic, management, or SACT options^8^ and associated values for the cost for each stage of disease.^9,10^ This problem is particularly relevant for economic evaluations of primary prevention and surveillance strategies, which save costs across the entire diagnostic-management-treatment pathway for cancers such as melanoma.

To enable analysts access to timely costs estimates to use in decision-analytic models, researchers can use structured exercises with experts to elicit the proportions of people accessing specified strategies for the diagnosis, management, and treatment of the disease.^11^ The analysts can then combine these proportions with published unit costs to calculate expected costs for each phase of cancer (diagnosis, management, SACT) and stage of cancer, such as melanoma. Unit costs are publicly available for all melanoma treatments. The exception to this are unit costs for SACT that NICE has appraised and deemed not cost-effective at the publicly available price and, hence, the NHS now purchases at a (confidential) reduced price.

There are published diagnosis, management, and SACT guidelines available from national bodies, such as NICE.^8^ However, these guidelines offer myriad options and do not provide overviews of commonly used diagnosis and subsequent management and treatment pathways. Estimates of the proportion of individuals out of a total population offered each option are also not available. By characterizing the various options for diagnosis, management, and prescription of SACT using a flow diagram (schematic) to represent alternative pathways, it is possible to provide a way of summarizing an evidence base with substantial variability. Such schematics can then form the basis of an elicitation exercise to estimate the costs of diagnosing, managing, and treating a cancer, such as melanoma, by stage of disease. Crucially, this approach also identifies the degree of uncertainty around the mean estimates of cost. The aim of this study was to illustrate how a simple structured expert elicitation exercise can be used to provide timely estimates of the costs of treating an exemplar cancer (melanoma) for each stage of the disease. This aim was met by 3 distinct objectives: describe examples of common approaches for the diagnosis, management, and use of SACT to summarize the many options potentially available to people with melanoma; elicit the proportion of people following specified routes through these diagnosis, management, and SACT options; calculate the costs of diagnosis, management, and SACT by stage of disease, also taking the uncertainty in these estimates into account.

## Methods

We developed an expert elicitation exercise to estimate the proportions of people using available options for the diagnosis, management, and prescription of SACT. The exercise was produced using Microsoft Excel.^12^ Expert elicitation, more generally, offers a suite of methods that can be used to collect judgments (or values) from groups of individuals defined as “an expert” in a specific topic.^13^ We report the design, conduct, and analysis of this expert elicitation exercise according to published reporting guidelines (see Appendix 1).^11^ The estimates of proportions were then combined with published unit costs to calculate the total costs for diagnosing, managing, and treating melanoma by stage of disease.

### Identifying Relevant Experts

The relevant experts used in this study were defined as people who diagnosed, managed, or treated people with melanoma following referral by a general practitioner (GP) to the hospital. We did not include GPs as experts in this study. This decision was confirmed to be appropriate by the experts who did take part in this study.

Experts, defined as consultant dermatologists, surgeons, or oncologists who diagnose and treat people experiencing melanoma, were invited to complete the expert elicitation exercise relevant to their specialty. A total of 61 experts (28 dermatologists, 10 surgeons, and 23 oncologists) were invited to take part in the exercise. We sought to capture possible regional differences in practice using purposive sampling of experts spread across the United Kingdom. We invited consultants in each specialty across different regions of the NHS to take part. Consultants were also asked to suggest the names of other experts in their specialty (sampling using “snowballing”).

### Defining Approaches for Diagnosis, Primary Surgical Management, and Use of SACT

We identified possible diagnostic strategies, management, and SACT options for melanoma by stage of disease from NICE guidelines^8^ and Cancer Research UK treatment information.^14^ We described and then drew (using schematics) pathways, each separately corresponding to options for diagnosis; management and treatment of the primary tumor encompassing lymph node dissection if required (primary management); and prescription of possible SACT, including genetic testing to identify eligibility for specific therapies (see Appendix 2). We refined each schematic based on feedback from an expert representing a relevant specialism (dermatology, surgery, oncology). None of the experts, after the pilot phase of this study, suggested any changes to the schematics (see Appendix 2).

### Structured Expert Elicitation Exercise

There is no agreed-upon format for an expert elicitation exercise to ask experts to estimate proportions together with a measure of the uncertainty around that estimate. Soares et al. identified a number of options, but this “good practice” report did not recommend a specific method.^15^ We chose to start by piloting 2 methods suggested within the Sheffield Elicitation Framework^16^ (SHELF): the quartile method and the roulette (also called “histogram”) method.^16^ These approaches are each described in detail on the Web site (Sheffield Elicitation Framework). One consultant from each specialty (dermatology, surgery, oncology) piloted the exercises and advised how to adapt them, including the question format. The pilot study identified that the 3 experts found the 2 possible SHELF methods for eliciting proportions (roulette and quartile) difficult to complete. For this reason, we developed our own structured expert elicitation exercise with a focus on making the questions simple and easy to complete. This exercise was piloted by 2 experts. We now describe this new method comprising distinct steps: estimating the point estimate of each proportion, estimating the range around this point estimate, and reporting the degree of certainty in the estimated proportion.

Each expert completed the exercise in Excel individually with the researcher. The researcher first asked each expert for their best estimate of each proportion. For each proportion, the expert was then asked to state their estimate for the lowest and highest plausible values. Then the expert was asked to express their view of the extent of certainty in their best estimate (not certain, moderately certain, very certain).

While completing the exercise, using a function in Excel, a scaled (4-parameter) beta distribution was fit to each expert’s answers. An excellent description of the 4-parameter beta distribution is provided by Wang.^17^ To calculate the beta distributions, we used the expert’s best estimate as the mean ( $\mu_{4}$ ). The lowest and highest plausible values were used as the lower and upper limits (max and min, respectively). We assumed precision (Φ) values for the expressed extent of certainty: 5 for not certain, 10 for moderately certain, and 20 for very certain. We used these answers to calculate the parameters of the 4-parameter beta ( $\alpha_{4}$ and $\beta_{4}$ ) distribution using equations 1 and 2.

(1) $$\alpha_{4}=\frac{\mu_{4}\times \Phi }{\max-\min}$$

(2) $$\beta_{4}=\Phi -\alpha_{4},$$

The results describing this distribution were then displayed (in “real time”) using the probability density function. We asked experts whether the displayed results conformed with their belief about the proportion of interest. If they did not, we asked the expert to adjust their answers until they were content with the estimated value for each proportion.

### Data Collection

**Figure 1:**
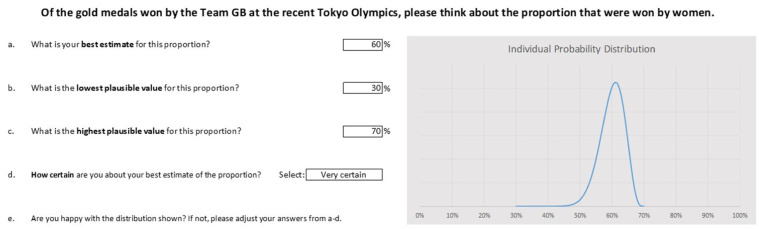
Practice question for the expert elicitation exercise.

We conducted the expert elicitation exercise over video call using Zoom. One researcher facilitated the exercise, leading the expert through each of the 4 sections, namely, defining options according to their specialty (diagnosis, management, SACT), an example question to familiarize them with the question format (Figure 1), the questions to elicit the proportions of interest, and experts’ personal and professional information. Each expert verified the diagnostic, management, or SACT options corresponding to their specialty and then estimated the related proportions.

### Analyzing Expert Values

Following data collection from each expert, the individual estimates were pooled to generate a group estimate with measure of variation. A sensitivity analysis was used to understand the impact of using a different method to pool the results.

We synthesized the estimates within each specialty (dermatology, surgery, oncology) using mathematical aggregation methods. The main analysis for pooling used random-effects meta-analysis to combine estimates. Random-effects meta-analysis incorporated between-expert variability in estimates of the proportions. This was done to capture any variation that may exist because of regional or center-specific difference in practice. We calculated the variance of the 4-parameter beta distribution that we elicited ( $\sigma_{4}^{2}$ ) using equation 3:

(3) $$\sigma_{4}^{2}=\frac{\alpha_{4}\beta_{4}(\max-\min)}{{\left(\alpha_{4}-\beta_{4}\right)}^{2}(\alpha_{4}+\beta_{4}+1)}$$

Then, we identified the 2-parameter beta distributions with mean and variance matching the 4-parameter betas using the method of moments (see equations 4 and 5)^18^:

(4) $$\;\alpha_{2}=(\frac{1-\mu_{4}}{\sigma_{4}^{2}}-\frac{1}{\mu_{4}})\times \mu_{4}^{2}$$

(5) $$\;\beta_{2}=\alpha_{2}(\frac{1}{\mu_{4}}-1)$$

We interpreted $\alpha_{2}$ as the number of people receiving a diagnostic, management, or SACT option in a hypothetical trial. The $\beta_{2}$ was interpreted as the proportion not receiving it. The sample size of the hypothetical trial ( $\alpha_{2}+\beta_{2})$ is proportional to the expert’s certainty.

To calculate a pooled estimate of each distribution across experts, we used the Freeman–Tukey (double arcsine) transformation^19^ for each hypothetical trial. These individual estimates were combined using random-effects meta-analysis. We converted the mean of the combined Freeman–Tukey statistic back to a proportion using the inverse of the Freeman–Tukey transformation. The variance in the mean proportion was calculated using delta methods.^20^ We then used the method of moments to calculate a 2-parameter beta distribution for the combined proportion.

In a sensitivity analysis, we used linear pooling to provide an alternative approach to combining the estimates. To perform linear pooling, we used simulation to sample from a 2-part model. First, we sampled an expert. Then we sampled a value from the 4-parameter beta provided by that expert. We computed the mean and variance of the samples from the 2-part process and used the method of moments to fit a beta distribution to them.

We tested the face validity of the proportions that experts estimated by comparing them with assumptions made in economic modeling developed to support NICE’s melanoma care guidelines,^8^ where possible.

### Sources of Unit Costs

Published sources were used to identify the relevant unit costs for diagnostics, surgical treatments, SACT, and staff time (see Appendix 3). The published unit costs were multiplied by estimates of the volume of service use for each option (diagnosis, management, SACT) by stage of disease. This approach was used to calculate the mean total costs by stage of melanoma.

We sourced hourly costs for staff time from Personal Social Services Research Unit estimates.^21^ Monthly costs for SACT (based on list prices) were taken from NICE melanoma care guidelines.^8^ Because all SACT are available only to the NHS with confidential discounts (patient access schemes), we undertook sensitivity analyses in which we reduced list prices by arbitrary amounts of 20%, 40%, and 60%. We used health care resource groups (HRGs) in the NHS 2024-25 Cost Collection^22^ to identify unit costs for health care services. For surgical management options, we consulted a clinical coder to find procedure (OPCS) codes. We then mapped OPCS codes to HRGs using NHS Digital’s 2024-25 Grouper software to identify the appropriate HRG code(s).^23^

### Calculating Costs

We aimed to estimate a mean total expected cost by stage of melanoma. We calculated the mean costs for each diagnostic, management, or SACT option represented in each schematic by multiplying the unit costs for consumables by the quantities used. We made assumptions, using input from the clinicians in the study team, about staff time spent on each appointment. The NICE melanoma guidelines^8^ informed assumptions about the number of follow-up appointments and months receiving SACT. We assumed that people who progressed to a later stage of melanoma during follow-up attended half the number of follow-up appointments. For HRGs that vary by anatomical site, we based weighted means on a study reporting the distribution of melanoma sites.^24^

To calculate the proportion of people offered each diagnostic, management, or SACT option for each stage of disease, we combined the elicited proportions from individuals. We used conditional logic to incorporate the probability of having reached the relevant point on the pathway. We multiplied the unit cost (diagnosis, management, SACT) with these proportions to estimate the mean total expected costs by melanoma stage and the variation (range) around this mean expected cost.

## Results

Of the invited 61 experts, 16 (26%) completed the expert elicitation exercise. The sample comprised 7 dermatologists, 5 oncologists, and 4 surgeons (July 2022–February 2023).

### Diagnosis, Primary Surgical Management, and Treatment Options

Based on the experiences of the experts included in this exercise, we defined possible options for the diagnosis, management, and prescribing of SACT for people with melanoma (see Appendix 2). For the available SACT, we were advised to group single-agent immunotherapy (nivolumab or pembrolizumab) and targeted therapy combinations (encorafenib with binimetinib and dabrafenib with trametinib). Table 1 shows the relevant questions to elicit the proportions of people with melanoma having each of the available options (see Appendix 2). Based on the advice of the experts, we excluded uncommon therapies (radiotherapy, electrochemotherapy, isolated limb perfusion, talimogene laherparepvec, and topical imiquimod) and uncommon surgical treatments (for example, resection of satellite and in-transit metastases).

**Table 1:** Questions Used in the Expert Elicitation Exercise to Understand the Proportions of Patients Offered Each Diagnostic, Primary Management, or Systemic Anticancer Therapy Option with Results

Question^a^	Main Analysis (Using Random-Effects Meta-Analysis)					Sensitivity Analysis (Using Linear Pooling)	
	Mean Estimate (Standard Error)	Distribution	Tau^2b^	I^2c^	*P* ^d^	Mean Estimate Standard Error)	Distribution
Diagnosis							
Q1: Of the people you examine for a clinically suspicious skin lesion, please think about the proportion who have clinical melanoma with palpable nodes	0.02 (0.01)	Beta (a = 15.07 b = 752.71)	0.01	0.95	<0.001	0.03 (0.04)	Beta∼ (a = 0.47 b = 16.33)
Q2: Of the people who do not have palpable nodes, please think about the proportion who receive a skin biopsy performed by a dermatologist or GP with special interest	0.32 (0.08)	Beta∼ (a = 9.63 b = 20.2)	0.22	0.98	<0.001	0.33 (0.24)	Beta∼ (a = 0.95 b = 1.91)
Q3: Of the people you see who receive a skin biopsy performed by a dermatologist or GP with special interest, please think about the proportion who receive an excision biopsy	0.92 (0.03)	Beta∼ (a = 94.12 b = 7.95)	0.06	0.95	<0.001	0.9 (0.07)	Beta∼ (a = 13.22 b = 1.42)
Q4: Of the people you see who receive a skin biopsy performed by a dermatologist or GP with special interest, please think about the proportion who have a diagnosis of melanoma confirmed by a histopathologist	0.43 (0.13)	Beta∼ (a = 5.81 b = 7.63)	0.47	0.99	<0.001	0.44 (0.22)	Beta∼ (a = 1.9 b = 2.41)
Q5: Of the people you see who have a diagnosis of melanoma confirmed by a histopathologist, please think about the proportion who are referred to a specialist nurse followed by surgery	0.95 (0.02)	Beta∼ (a = 152.7 b = 8.77)	0.03	0.88	<0.001	0.94 (0.04)	Beta∼ (a = 31.92 b = 2.09)
Management							
Q1: Please think about the proportion of people who receive imaging for stage 1b-2 melanoma	0.13 (0.06)	Beta∼ (a = 3.73 b = 25.78)	0.13	0.99	<0.001	0.16 (0.12)	Beta∼ (a = 1.18 b = 6.42)
Q2: Please think about the proportion of the people with stage 1b-2 melanoma who receive wide local excision with direct closure	0.69 (0.07)	Beta∼ (a = 26.03 b = 11.9)	0.10	0.96	<0.001	0.68 (0.17)	Beta∼ (a = 4.44 b = 2.14)
Q3: Of those who don’t receive direct closure, please think about the proportion of the people with stage 1b-2 melanoma who receive wide local excision with a split/full thickness graft	0.23 (0.11)	Beta∼ (a = 3.04 b = 10.15)	0.27	0.99	<0.001	0.25 (0.17)	Beta∼ (a = 1.39 b = 4.16)
Q4: Please think about the proportion of people with stage 1b-2 melanoma upstaged to 3 following sentinel lymph node biopsy	0.2 (0.03)	Beta∼ (a = 48.68 b = 190.63)	0.02	0.98	<0.001	0.21 (0.03)	Beta∼ (a = 29.96 b = 116.17)
Q5: Please think about the proportion of people with stage 1b-2 melanoma upstaged to 3 during follow-up visits	0.14 (0.04)	Beta∼ (a = 10.49 b = 63.08)	0.05	0.97	<0.001	0.15 (0.06)	Beta∼ (a = 4.69 b = 27.63)
Q6: Please think about the proportion of people with macroscopic stage 3 melanoma upstaged to 4 following imaging	0.33 (0.1)	Beta∼ (a = 6.49 b = 13.27)	0.18	0.97	<0.001	0.34 (0.14)	Beta∼ (a = 3.73 b = 7.33)
Q7: Please think about the proportion of people with microscopic stage 3 who receive lymph node dissection	0.05 (0.02)	Beta∼ (a = 9.37 b = 173.33)	0.02	0.96	<0.001	0.06 (0.03)	Beta∼ (a = 3.38 b = 58.15)
Q8: Please think about the proportion of people with microscopic stage 3 melanoma upstaged to 4 during follow-up visits	0.19 (0.02)	Beta∼ (a = 88.81 b = 372.75)	0.01	0.73	0.010	0.21 (0.06)	Beta∼ (a = 8.52 b = 31.59)
Q9: Please think about the proportion of people with stage 4 melanoma receiving excision of solitary metastasis	0.24 (0.16)	Beta∼ (a = 1.45 b = 4.69)	0.55	0.99	<0.001	0.27 (0.25)	Beta∼ (a = 0.56 b = 1.51)
Systemic anticancer therapies (SACT)							
Q1: Out of people who you treat for stage III melanoma, please think about the proportion who are offered genetic testing to guide treatment selection	0.96 (0.01)	Beta∼ (a = 229.87 b = 9.55)	0.01	0.78	0.001	0.96 (0.04)	Beta∼ (a = 27.52 b = 1.3)
Q2: Out of the people who you treat for stage III melanoma, please think about the proportion who receive adjuvant systemic therapy	0.81 (0.03)	Beta∼ (a = 163.75 b = 39.45)	0.02	0.76	0.002	0.8 (0.06)	Beta∼ (a = 31.86 b = 7.97)
Q3: Out of the people who you treat for BRAF-negative (wild-type) stage III melanoma, please think about the proportion who are prescribed single agent adjuvant therapy (nivolumab or pembrolizumab)	0.87 (0.05)	Beta∼ (a = 44.52 b = 6.62)	0.08	0.93	<0.001	0.86 (0.11)	Beta∼ (a = 7.86 b = 1.29)
Q4: Out of the people who you treat for BRAF-positive stage III melanoma, please think about the proportion who are prescribed single agent adjuvant therapy (nivolumab or pembrolizumab)	0.22 (0.18)	Beta∼ (a = 0.89 b = 3.23)	0.96	1.00	<0.001	0.29 (0.32)	Beta∼ (a = 0.28 b = 0.69)
Q5: Out of the people who you treat for stage IV melanoma, please think about the proportion who would be offered genetic testing to guide treatment selection	0.95 (0.01)	Beta∼ (a = 205.45 b = 10.84)	0.01	0.73	0.005	0.96 (0.04)	Beta∼ (a = 23.51 b = 1.03)
Q6: Out of the people who you treat for BRAF-negative (wild-type) stage IV melanoma, please think about the proportion who are prescribed single agent immunotherapy (nivolumab or pembrolizumab)	0.48 (0.09)	Beta∼ (a = 15.73 b = 17.23)	0.14	0.98	<0.001	0.48 (0.14)	Beta∼ (a = 5.52 b = 5.98)
Q7: Out of the people who you treat for BRAF-positive stage IV melanoma, please think about the proportion who are prescribed targeted therapy (encorafenib in combination with binimetinib or dabrafenib in combination with trametinib) as a first line treatment	0.13 (0.02)	Beta∼ (a = 26.71 b = 174.7)	0.02	0.82	<0.001	0.14 (0.06)	Beta∼ (a = 4.94 b = 30.37)
Q8: Out of the people who you treat for BRAF-positive stage IV melanoma with immunotherapy, please think about the proportion who are prescribed single agent immunotherapy (nivolumab or pembrolizumab) as opposed to combination therapy	0.34 (0.14)	Beta∼ (a = 3.41 b = 6.6)	0.44	0.99	<0.001	0.36 (0.25)	Beta∼ (a = 0.93 b = 1.66)
Q9: Out of the people who you treat for BRAF-negative (wild-type) stage IV melanoma, please think about the proportion who are prescribed ipilimumab as a second line therapy	0.29 (0.05)	Beta∼ (a = 22.35 b = 54.75)	0.06	0.95	<0.001	0.31 (0.15)	Beta∼ (a = 2.5 b = 5.57)
Q10: Out of the people who you treat for BRAF-positive stage IV melanoma who received targeted therapy as a first line treatment, please think about the proportion who are prescribed 2nd line immunotherapy	0.35 (0.14)	Beta∼ (a = 3.98 b = 7.24)	0.39	0.98	<0.001	0.37 (0.21)	Beta∼ (a = 1.53 b = 2.6)
Q11: Out of the people who you treat for BRAF-positive stage IV melanoma who are prescribed second-line immunotherapy, please think about the proportion who receive single agent therapy (pembrolizumab or nivolumab)	0.68 (0.09)	Beta∼ (a = 18.74 b = 8.87)	0.16	0.98	<0.001	0.67 (0.19)	Beta∼ (a = 3.41 b = 1.68)
Q12: Out of the people who you treat for BRAF-positive stage IV melanoma who received immunotherapy as a first line treatment, please think about the proportion who are prescribed second-line treatment using targeted therapy (encorafenib + binimetinib or dabrafenib + trametinib)	0.64 (0.12)	Beta∼ (a = 10.21 b = 5.78)	0.28	0.99	<0.001	0.61 (0.24)	Beta∼ (a = 1.84 b = 1.17)
Q13: Out of the people who you treat for stage IV melanoma, please think about the proportion are referred for palliative care at any stage in the pathway	0.93 (0.02)	Beta∼ (a = 136.07 b = 10.36)	0.02	0.87	<0.001	0.84 (0.23)	Beta∼ (a = 1.22 b = 0.23)

GP, general practitioner.

a The “question” numbers reference the respective diagrams shown in Appendix 2.

b Tau^2^ is the variance of the between-expert distribution estimated in the random-effects model.

c I^2^ is the proportion of observed variance that is attributable to between-expert heterogeneity.

d *P* is derived from Cochran’s *Q* test, estimating the extent to which the data are consistent with a null hypothesis of homogeneous expert values.

### Expert Elicitation of Proportions

**Figure 2:**
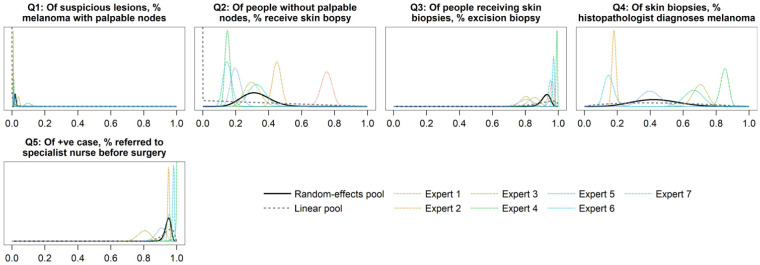
Estimated proportions for the diagnosis of melanoma. *Q* relates to the question number shown in the diagram in Appendix 2.

Figures 2 through 4 show individual and pooled distributions of the proportions of people receiving specific diagnostic tests, management options, and SACT options, respectively. Table 1 presents the proportions that were elicited for each question and the characteristics of the combined distributions for each proportion.

**Figure 3:**
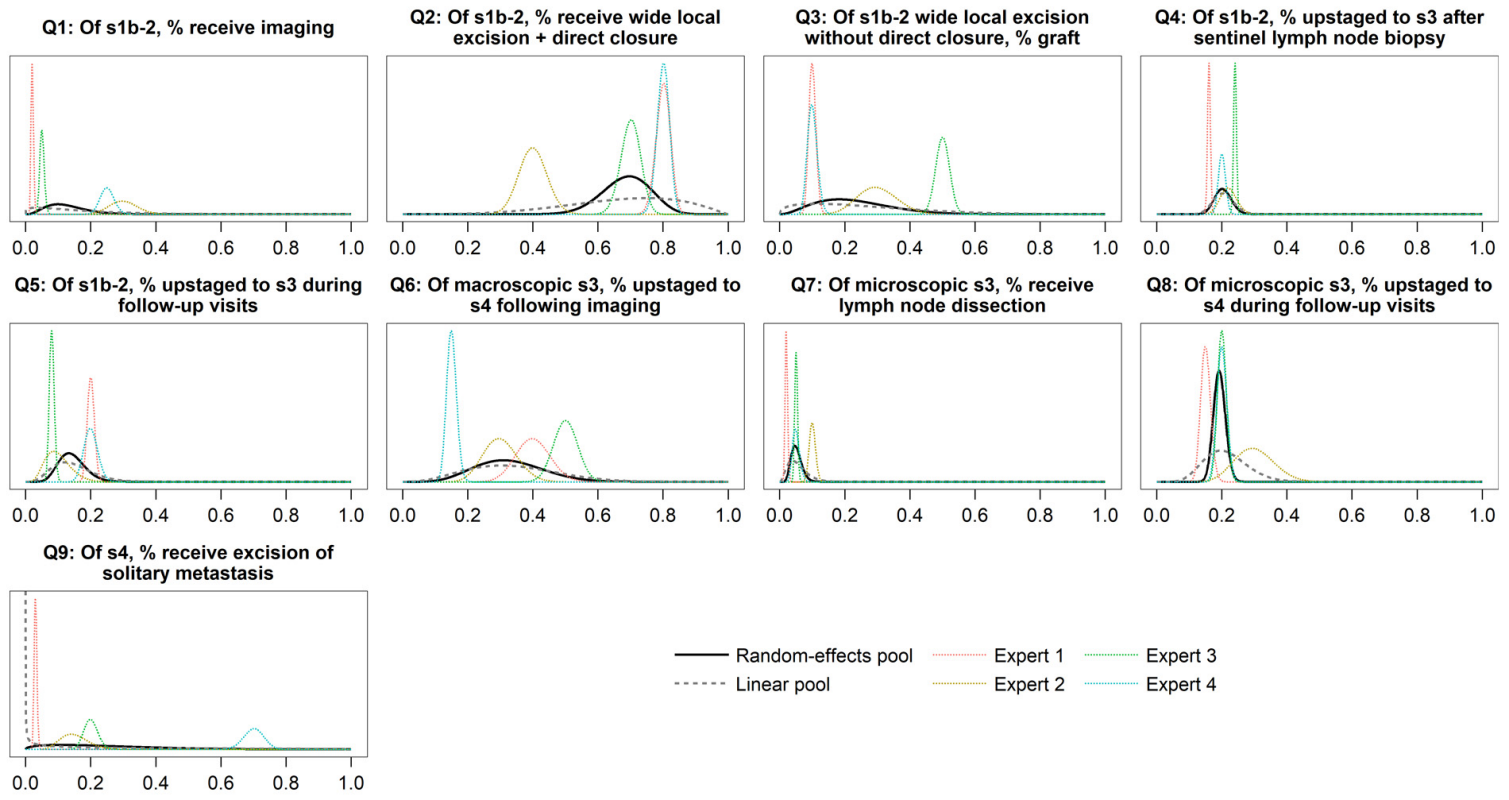
Estimated proportions for the primary surgical management of melanoma. *Q* relates to the question number shown in the diagram in Appendix 2.

**Figure 4:**
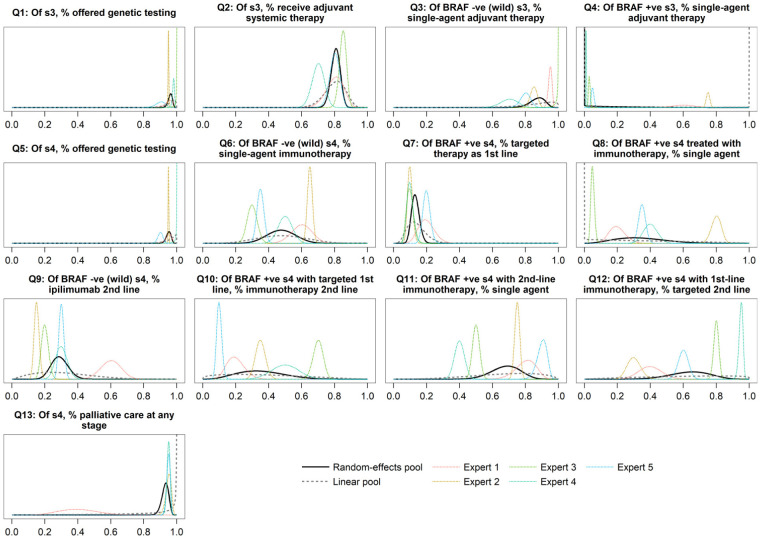
Estimated proportions for the use of systemic anticancer therapies for melanoma. *Q* relates to the question number shown in the diagram in Appendix 2. IT, immunotherapy; TT, targeted therapy.

There was extensive variability (within-expert and between-expert variability) in the estimated proportions receiving skin biopsy for suspicious skin lesions and histological diagnosis of melanoma. For the primary tumor management pathway, there was substantial variability in the estimated proportions: receiving wide local excision for stage 1b-2 melanoma, who were upstaged following imaging for macroscopic stage 3 melanoma, and who had solitary metastases excised for stage 4 melanoma.

Within the available SACT options, variability in which medicines were selected was especially high for the proportion of people offered immunotherapy (single-agent nivolumab or pembrolizumab). The following estimated proportions were highly variable for people with BRAF-positive stage 4 melanoma who received a single agent if they received immunotherapy, second-line immunotherapy following targeted therapy, and targeted therapy as second line following immunotherapy.

In general, experts’ estimates displayed considerable heterogeneity. The *I*^2^ statistic indicated that between-expert variability accounted for more than 75% of overall variability in the pooled distributions (using random-effects meta-analysis). There were 2 exceptions for the proportion of people experiencing microscopic stage 3 melanoma upstaged to stage 4 during follow-up visits and stage 4 melanoma offered genetic testing. The chi-squared test for between-expert heterogeneity was statistically significant at the 5% level for all proportions. Using linear pooling rather than random-effects meta-analysis resulted in similar means but larger standard errors, suggesting more variability.

### Total Costs

Table 2 presents mean total expected costs for diagnosis, management, and SACT calculated using the relevant unit costs (Appendix 3). The mean cost for diagnosing that a suspicious skin lesion is not melanoma was £424, including a visit to the GP and dermatologist with dermoscopy. There was no range (95% confidence interval) presented for the mean diagnostic cost because both the options for diagnosis attract the same HRG, generating the same unit cost. Macroscopic stage 3 melanoma incurred a mean diagnosis cost of £484, as this requires a biopsy but no dermoscopy. For all other cases of melanoma, the mean diagnosis cost was £699 including the cost of biopsy and dermoscopy. Experts provided different estimates for the proportion of people receiving excisional biopsy, but this did not change the mean cost because all types of biopsy attract the same HRG code.

**Table 2:** Diagnosis, Primary Management, and Systemic Anticancer Therapy Costs by Melanoma Stage

Item	Unit Cost (£; 2024/2025)	Expected Mean Cost, £ (95% Confidence Interval)							
		Lesion Not Melanoma		Stage 0	Stage 1a	Stage 1b/2	Stage 3		Stage 4
		Diagnosed in Clinic	Requiring Biopsy				Microscopic	Macroscopic	
Diagnosis									
GP appointment	45	45	45	45	45	45	—	45	—
Dermatology appointment	165	165	165	165	165	165	—	165	—
Dermoscopy	215	215	215	215	215	215	—	—	—
Excision biopsy	215	—	198 (186–207)	198 (186–207)	198 (186–207)	198 (186–207)	—	215	—
Shave/punch biopsy	215	—	17 (7–29)	17 (7–29)	17 (7–29)	17 (7–29)	—	—	—
Histopathology	60	—	60	60	60	60	—	60	—
Diagnosis total		424	699 (699–699)^a^	699 (699–699)^a^	699 (699–699)^a^	699 (699–699)^a^	—	484	—
Management									
Specialist nurse	57	—	—	54 (52–56)	54 (52–56)	54 (52–56)	—	54 (52–56)	—
Wide local excision direct closure	215	—	—	148 (114–176)	148 (114–176)	148 (114–176)	—	—	—
Wide local excision graft	264	—	—	19 (4–44)	19 (4–44)	19 (4–44)	—	—	—
Wide local excision flap	1,650	—	—	397 (200–626)	397 (200–626)	397 (200–626)	—	—	—
1× follow-up after wide local excision	165	—	—	165	—	—	—	—	—
2× follow-up after wide local excision	329	—	—	—	329	—	—	—	—
Sentinel lymph node biopsy	2,970	—	—	—	—	2,970	—	—	—
Imaging (brain MRI + PETCT or CT of thorax/abdomen/pelvis)	360	—	—	—	—	45 (13–93)	360	360	—
Lymph node dissection	1,644	—	—	—	—	—	84 (40–144)	1,644	—
Histopathology	60	—	—	—	—	—	3 (1–5)	60	—
Complete 5-y follow-up (stage 1b/2)	1,481	—	—	—	—	1,012 (896–1,119)	—	—	—
Upstage during 5-y follow-up (stage 1b/2)	741	—	—	—	—	84 (42–137)	—	—	—
Complete 5-y follow-up (stage 3)	2,633	—	—	—	—	—	2,127 (2,029–2,221)	1,431 (974–1,824)	—
Upstage during 5-y follow-up (stage 3)	1,316	—	—	—	—	—	253 (206–302)	170 (112–231)	—
Management total		0 (0–0)	0 (0–0)	782 (611–981)	946 (776–1,145)	4,729 (4,532–4,944)	2,827 (2,757–2,904)	3,719 (3,213–4,150)	0 (0–0)
Systemic anticancer therapies (SACT)									
Genetic test (BRAF)	75	—	—	—	—	—	58 (54–62)	58 (54–62)	71 (69–73)
Single-agent nivolumab/pembrolizumab	48,586	—	—	—	—	—	25,738 (21,119–32,137)	25,738 (21,119–32,137)	20,288 (13,798–27,153)
Nivolumab + ipilumimab	57,837	—	—	—	—	—	4,059 (1,684–7,300)	4,059 (1,684–7,300)	31,203 (22,983–38,980)
Dabrafenib + trametinib	172,032	—	—	—	—	—	35,434 (15,024–46,242)	35,434 (15,024–46,242)	—
Targeted therapy (dabrafenib + trametinib or encorafenib + binimetinib)	178,607	—	—	—	—	—	—	—	7,667 (5,166–10,543)
6× follow-up (stage 4 only)	987	—	—	—	—	—	—	—	987
Single-agent nivolumab/pembrolizumab (second line)	48,586	—	—	—	—	—	—	—	505 (153–1,013)
Nivolumab + ipilimumab (second line)	57,837	—	—	—	—	—	—	—	284 (77–645)
Ipilimumab (second line)	210,437	—	—	—	—	—	—	—	41,149 (27,466–56,381)
Targeted therapy (dabrafenib + trametinib or encorafenib + binimetinib; second line)	178,607	—	—	—	—	—	—	—	31,915 (19,747–42,672)
6× follow-up (stage 4)	987	—	—	—	—	—	—	—	987
SACT total		0 (0–0)	0 (0–0)	0 (0–0)	0 (0–0)	0 (0–0)	65,289 (50,581–74,521)	65,289 (50,581–74,521)	134,065 (115,787–152,938)

CT, computed tomography; GP, general practitioner; MRI, magnetic resonance imaging; PET, positron emission tomography.

a No variation as the 2 alternative diagnostic options have the same unit cost.

The mean management costs (and 95% confidence intervals based on sampling from random-effects models) were £782 (£611 to £981) for stage 0, £946 (£776 to £1,145) for stage 1a, £4,729 (£4,532 to £4,944) for stage 1b/2, £2,827 (£2,757 to £2,904) for microscopic 3, and £3,719 (£3,213 to £4,150) for macroscopic 3. Higher management costs for later stages were driven by greater numbers of follow-up appointments and costly procedures such as sentinel lymph node biopsy and lymph node dissection. People experiencing stage 1b-2 melanoma incurred higher costs than those experiencing macroscopic stage 3 melanoma because sentinel lymph node biopsy is more costly than lymph node dissection. Management costs for macroscopic stage 3 melanoma were most uncertain, reflecting substantial between-expert heterogeneity.

Most of the total cost for people experiencing stage 3 and 4 melanoma was driven by SACT costs. The mean treatment costs were considerably higher than diagnosis and management costs. Assuming list-price acquisition costs, the mean SACT costs were £65,289 (£50,581 to £74,521) for stage 3 and £134,065 (£115,787 to £152,938) for stage 4. Using SACT for stage 3 melanoma required no more than 1 y of treatment, typically with 1 line of nivolumab or pembrolizumab. In contrast, using SACT for stage 4 melanoma continues until progression or for a minimum length of 2 y and commonly involves multiple lines of treatment including ipilimumab or targeted therapy. The mean total expected costs for SACT for stage 3 and stage 4 melanoma were uncertain, reflecting between-expert heterogeneity in the estimates of proportions receiving the more costly agents (targeted therapies, second-line ipilimumab).

Using the results from the linear pooling in the sensitivity analysis suggested that the estimated mean costs were considerably more uncertain (Appendix 4), because this method produces more variable pooled estimates for each proportion informing the costs.

We also looked at the total expected costs calculated for each individual expert’s answers (see Appendix 5). This analysis suggested that costs associated with diagnosis did not vary according to dermatologist, because the treatments that they estimated that different proportions received attracted the same cost codes. Costs for management varied most for stage 1b-2 and macroscopic stage 3 melanoma. This variability was driven by the factors influencing management costs (receiving flap repair after wide local excision and upstaged from macroscopic stage 3 after imaging). The costs for SACT varied considerably between experts’ answers for both stages 3 and 4, but especially for stage 4. The costs for using SACT in stage 4 melanoma appeared to vary more in the per-expert analysis than in the main analysis based on random-effects meta-analysis pooling.

Assuming different price reductions for the unit cost of SACT (see Appendix 6), the overall mean costs reduced by about the same applied proportional reduction. This proportionate reduction was seen because all relevant agents are subject to patient access schemes. Genetic testing and follow-up costs, which represent a fraction of the SACT costs, were not affected.

## Discussion

This study has illustrated a simple expert elicitation method to generate estimates of the costs associated with the diagnosis, management, and use of SACT for melanoma by stage of disease. We did not consider palliative care because this would require developing a further pathway or making existing pathways overly complex.

Our proposed method can be used when linked datasets to calculate such costs are not readily available. Because experts found published formats for eliciting proportions difficult, we developed a new exercise to elicit a best estimate, upper and lower plausible values, and a qualitative level of certainty in these estimates. The suggested method could be used in other applications when it is necessary to estimate a proportion, with a measure of variation around the mean estimate, for use in a decision-analytic model.

We focused on melanoma as an exemplar. The experts verified that the schematics we produced representing options for the diagnostics, management, and use of SACT for melanoma reflected their respective practices. These experts provided plausible estimates of the proportions of people experiencing melanoma who received specified diagnostic tests, management options, and SACT as described by each relevant schematic. Pooled cost estimates for each option had face validity, as the estimated values for less severe stages of disease (stage 0 to 2) were less than the more advanced stages of disease (3 or 4).

Some elicited estimates for proportions displayed substantial heterogeneity, which resulted in variability in the calculated costs. This variation was especially apparent for SACT costs relating to targeted treatment and second-line ipilimumab. Looking at the individual expert estimates suggested that geographical variability in the costs associated with SACT for treating stage 4 melanoma could be pronounced, with wider variation than observed in the confidence intervals of our probabilistic analysis. The aim of our probabilistic analysis was to capture uncertainty around expected values for the setting of interest. It was therefore unsurprising that we were more confident in the range of values the total mean estimated cost could possibly take than in the spread of individual estimates. Nevertheless, users of these values should give thought to the meaning of a random-effects distribution in this context; we suggest that similar considerations apply here to those discussed in relation to relative treatment effects.^25^

The costs associated with managing the primary tumor (management) increased with more severe stages of melanoma, which require more follow-up appointments and procedures such as sentinel lymph node biopsy and lymph node dissection. The use of SACT was more costly than diagnosis or management. The cost of treating stage 4 melanoma far exceeded the costs of treating stage 3 melanoma, reflecting the longer duration of treatment for people with more severe disease.

### Strengths and Limitations

This study has developed a method using a simple question format for expert elicitations of proportions. We summarized the available options for the diagnosis, management, and prescription of SACT for the treatment melanoma by stage of disease in accordance with national guidelines. We illustrated how you can use this approach to calculate the total costs for diagnosing, managing, and treating melanoma for each stage of melanoma in the absence of timely and accessible linked datasets.

Although experts were more comfortable providing upper and lower plausible values in the revised question format, the best use of these values to parameterize model inputs is not clear. Using them as confidence intervals would require fitting approximate models. Using them as bounds on a 4-parameter distribution allows exact modeling. However, the 2-parameter beta is a more natural choice for modeling the sampling distribution of a proportion. Furthermore, 4-parameter distributions would be difficult to synthesize without simulation. In our main analysis, we decided to approximate the 4-parameter beta distributions that we elicited with 2-parameter versions. The mean and variance of the 2-parameter distributions matched the originals.

In principle, between-expert heterogeneity should reflect true differences in practice due to geographical variation. We are confident that, as directed, experts interpreted the questions in terms of relevance for their geographical region. However, although we piloted the exercises, it is also plausible that between-expert heterogeneity reflected different interpretations of the questions. Each expert was required to understand the conditionality of their responses correctly. In principle, this should have meant they took previous branches in the pathway into account; however, it would explain some heterogeneity if, in practice, the implicit denominators in experts’ thought processes varied. It is also plausible that the observed variability in cost estimates may be a consequence of different degrees of knowledge on the part of experts.

To estimate overall costs, we sampled from the distributions corresponding to each question independently. This approach does not account for any potential correlation between the proportions specified by following one branch of the pathway; for example, services that offer a particular treatment early in the pathway may be more or less likely to offer a different treatment later. Such differences may feasibly exist because of geographical differences in practice, for example, between Scotland and England.

### Comparison with Other Literature

The NICE guidelines for managing stage 3 and 4 melanoma^8^ are based on economic evidence from an original decision-analytic model. For this model, the developers estimated costs for progression-free melanoma, post-progression, palliative care, and terminal care. The evidence for BRAF testing combined conditional probabilities to calculate single proportions of people receiving each first- and second-line treatment. However, the guidelines did not provide a clear description of the possible options and sequence of choices between alternative treatments along the diagnosis-management-treatment pathway. Nor do they suggest how many individuals within a population (the proportion) are offered each of the different options or captured potential variation around these estimates of proportions in a probabilistic way.

A study from Northern Ireland published in 2024 has mapped and costed a simple care pathway for skin cancers, including melanoma.^26^ This study based the proportions of people receiving each diagnostic strategy, management option, or SACT on performance measures from registry datasets supported with expert opinion. The authors estimated that the mean costs for managing a case of melanoma was £31,300 (or £26,315 assuming 75% utilization of systemic therapy regimens). These estimates are consistent with ours in terms of scale.

A number of published costs estimates for the treatment of melanoma are available, but none of these include costs associated with diagnosis or surgery. In 2018, Wilson and colleagues assumed that the cost of treating a stage 0 melanoma was £396 and a stage IV melanoma was £4,761. These estimates were based on now outmoded treatment options.^6^ The authors calculated melanoma treatment costs according to the NICE guidelines at the time, not including SACT. They also assumed that all people with the same stage of melanoma received the same options for management and treatment.^6^

In 2022, Buja and colleagues estimated melanoma costs by demographic and disease characteristics in Italy using registry data.^27^ Buja and colleagues reported that the cost of treating stage I melanoma was €889 (95% confidence interval: €686 to €1092) and stage IV was €33,890 (95% confidence interval: €23,436 to €44,343).^27^

Other authors have estimated melanoma care costs by stage of diagnosis, using the same decision-analytic model to simulate the main management and treatment options.^24,28^ In 2025, Mistry and colleagues^7^ reported numbers for treating stage 1 to stage IV melanoma by inflating the estimates provided by 2 previously published studies.^26,29^ Using this inflation-index approach, these authors reported treatment costs (£; 2023) for 4 stages of melanoma: £9,512 for stage I, £77,813 for stage II, £179,274 for stage III, and £213,801 for stage IV.^7^

Although the exact values of these other treatment costs did not match ours, all previous studies reflected our observation that mean costs rise at an accelerating rate for people diagnosed at later stages. Our expert elicitation exercise suggested that the proportions following each option considerably influenced estimated costs for surgery and treatments. Our estimated mean costs associated with SACT were lower than some of these published estimates at £5,289 for stage 3 and £134,065 for stage 4. We conducted our exercise in 2022 and 2023, and since then, there have been more new SACT options for treating melanoma. This illustrates the speed at which new treatment options for melanoma are introduced. Mistry and colleagues estimated costs by inflating older estimates rather using current SACT options. It is not, therefore, possible to say which of these widely different values are the correct costs of treating melanoma. This highlights the importance of using methods that not only estimate point estimates but also appropriately reflect uncertainty in the data.

Decision-analytic modelers have previously elicited proportions of interest using the SHELF question, which asks experts to estimate quartiles.^30,31^ Some analysts have found the method worked well, but experts required an extensive one-to-one training presentation before each exercise. Due to experts’ time constraints, we could not provide one-to-one training. Guthrie and colleagues^31^ used the quartile variant of SHELF methods to elicit proportions of interest from 6 experts but did not report experts’ understanding or experience of the exercise. Consistent with our observations when conducting the pilot study, Cope and colleagues^32^ previously observed the roulette method to be challenging to use in practice. Other authors have noted that the roulette method works best “when experts have a solid background in statistics,”^16^ which is likely to pose challenges in most applied cases.

### Implications

The schematics we produced to summarize the diagnosis, management, and prescription of SACT for each stage of melanoma may help inform the conceptualization of decision-analytic models used to evaluate strategies for early detection or prevention strategies. The distributions of service-use proportions and costs by melanoma stage that we report may help to populate these models and enable probabilistic sensitivity analysis. The estimated mean costs, and the observed variation around them, are especially useful in the evaluation of prevention and early detection strategies, as they provide estimates of the cost of diagnosing people at earlier stages of disease (stages 0 to 2) rather than stages 3 to 4.

The best way to elicit expert opinion about proportions (and associated uncertainty) remains unclear. SHELF methods are theoretically sound but have been observed to be difficult to use.^16,32^ We have presented an alternative method that experts found easier to complete. However, the method we propose may make it more difficult to subsequently fit a parametric model to estimates. After completing our study, we reflected how a simple—and potentially accurate—compromise may be to elicit a 2-parameter beta distribution by asking experts to provide a best estimate and visualize their uncertainty using a slider. In the “back end,” the slider could control the precision parameter of a 2-parameter beta. Researchers could interpret the best estimate as the mean and use this with the precision to calculate shape and scale parameters (α and β). This potential approach requires piloting in a follow-up study.

## Conclusion

We developed a method that uses a simple question format for use in an expert elicitation exercise estimating proportions and a measure of uncertainty, which balanced practical with theoretical considerations. The experience of completing and analyzing this exercise raised methodological questions about how best to elicit proportions from experts to inform cost estimates. This study has also highlighted that variability in observed cost estimates may be a consequence of experts’ subjective interpretation of elicitation questions and reflects uncertainty in their knowledge. In the future, decision-analytic models that evaluate early detection or prevention strategies for melanoma may use the cost estimates we have produced in this expert elicitation study. Caution is advised as these cost estimates were not validated against registry data. Any analyst using these costs estimates in a decision-analytic model must ensure that they are subject to robust sensitivity analysis to understand the impact of using different values on the observed results. In the long run, if and when access to the required diagnosis, management, and prescribing data becomes available, a subsequent study could offer an external validation of our elicited cost estimates.

## Supplemental Material

sj-docx-1-mpp-10.1177_23814683261451278 – Supplemental material for A Method for Estimating Resource Use and Costs when Empirical Data Are Unavailable: Expert Elicitation Study Using the Example of Melanoma Supplemental material, sj-docx-1-mpp-10.1177_23814683261451278 for A Method for Estimating Resource Use and Costs when Empirical Data Are Unavailable: Expert Elicitation Study Using the Example of Melanoma by Rob Hainsworth, Louisa Collins, Martin Eden, Adele Green, Paul Lorigan, Gabriel Rogers, Amber Salisbury and Katherine Payne in MDM Policy & Practice
