# Supplementary material for: A Method for Estimating Resource Use and Costs when Empirical Data Are Unavailable: Expert Elicitation Study Using the Example of Melanoma
Source: MDM Policy Pract. 2026 Jun 25;11(1):23814683261451278. doi: 10.1177/23814683261451278 (PMC13305569; doi:10.1177/23814683261451278)
Supplement: sj-docx-1-mpp-10.1177_23814683261451278 – Supplemental material for A Method for Estimating Resource Use and Costs when Empirical Data Are Unavailable: Expert Elicitation Study Using the Example of Melanoma [file sj-docx-1-mpp-10.1177_23814683261451278.docx]

**Appendices for: Estimating the costs of diagnosing and managing melanoma for use in cost-effectiveness analyses: an expert elicitation study**

List of Appendices

Appendix 1 – Reporting guidelines for expert elicitation studies

Appendix 2- Options for the diagnosis, primary management and prescription of systemic anti-cancer therapies for melanoma

Appendix 3 – Information used to calculate total costs

Appendix 4- Results of the sensitivity analysis based on linear pooling method for combining experts’ estimates

Appendix 5- Sensitivity analysis showing total costs calculated using responses for each individual expert

Appendix 6 - Sensitivity analysis showing total costs calculated using hypothetical values for the unit costs of systemic anti-cancer therapies (SACT)

### Appendix 1 – Reporting guidelines for expert elicitation studies

**[see** [**Reporting Guidelines for the Use of Expert Judgement in Model-Based Economic Evaluations - PubMed**](https://pubmed.ncbi.nlm.nih.gov/27364887/)**]**

| **Criterion** | **Description** | **Note** | **Section** |  |  |
| --- | --- | --- | --- | --- | --- |
| Research rationale | The need for using an expert elicitation exercise should be described | This should ideally include some reference to the design and conduct of systematic reviews to identify key input parameters for the decision–analytic model and a statement confirming that these reviews did not identify data relevant for the model-based economic analysis as specified | Introduction |  |  |
| Research problem | All uncertain quantities (model input parameters) that will be elicited should be described | In some instances, there may be a substantial number of uncertain quantities required, and a degree of ‘pre-selection’ will have occurred to identify a relevant subset. Clear justification for model parameters identified as key for the decision problem needs to be provided | Table 1 and Results, describing the approaches to diagnosis, primary management and use of systemic anti-cancer therapies |  |  |
| Measurement type of uncertain quantities | The rationale for the measure type of each uncertain quantity elicited should be described | The measurement type of uncertain quantities can be (but not limited to): scalar quantities (i.e. numbers); proportions (e.g. probabilities); ratios (e.g. odds, hazard); risk (e.g. relative); rate (e.g. mortality), etc. Some measures are easier to understand and elicit than others; thus, it is important to fully justify the selection of any measurement type | Introduction |  |  |
| Definition of an expert | The nature of the expert population should be described to clearly state what topic of expertise they represent and why | It is unlikely that a single expert will be sufficient and it is generally necessary to elicit judgement from a group of experts that were selected to represent the views of a larger population | Methods |  |  |
| Number of experts | The selection criteria and final number of experts recruited to provide expert judgement should be reported | Selection criteria need to be described in detail. There should be clear and specific pre-defined criteria used to identify how experts were selected and if/how their elicited quantities were used | Methods, Identifying relevant experts |  |  |
| Preparation | There should be clear reference made to a protocol that describes the design and conduct of the elicitation exercise | None | Methods, Structured expert elicitation exercise |  |  |
| Piloting | It should be clearly reported if the elicitation exercise process was piloted and a summary of any modifications made | The selection and number of experts used in the piloting process should be reported. Key aspects that may have required modification include: selection of experts; measure type and number of uncertain quantities to be elicited; training exercise; framing of the elicitation question; method of aggregation | Methods, Structured expert elicitation exercise |  |  |
| Data collection | The approach to collect the data should be reported | Data can be collected from individual experts or a group/s of experts. Collecting data from individual experts means that a mathematical aggregation process may need to be used. Collecting data from group(s) of experts means that behavioural aggregation methods may be used | Methods, Data collection and Analysing expert values |  |  |
| Administration | The mode of administering the elicitation exercise should be reported | Elicitation exercises can be conducted face-to-face or via the telephone and/or computer. In a limited number of situations it may be feasible to collect the data using a self-administered online or postal survey but this is unlikely to be successful in most instances. Both face-to-face and telephone data collection is likely to be supported by using a computer | Methods, Data collection |  |  |
| Training | The use of training materials should be reported and made available | This may include background training materials sent to the experts and/or training in the use of probabilities and nature of distributions. This document need to provide explanation of efforts made to prevent influencing experts’ knowledge and judgement. In practice, this recommendation will require a copy of the elicitation exercise to be included, which is likely to be presented as electronic supplementary material | Methods, Data collection |  |  |
| The exercise | The number and framing of questions used in the exercise should be reported and made available | This will require a copy of the elicitation exercise to be included, which is likely to be presented as electronic supplementary material | Table 1, Figure 1 and Appendix 2 |  |  |
| Data aggregation | The type of aggregation method (mathematical or behavioural) should be reported together with a description of the method or process used to aggregate the data | Mathematical aggregation (relevant when data were collected from multiple individual experts) can be conducted using a range of methods, for example: Bayesian methods; opinion pooling; Cooke’s method. ehavioural aggregation (relevant when data were collected from group(s) of experts) can be conducted using processes such as, for example: Delphi or Nominal Group technique | Methods, Analysing expert values |  |  |
| Measures of performance for data aggregation | The processes followed to estimate measures of performance (calibration/information) for data aggregation need to be fully described | Calibration is the process of measuring the performance of experts by comparing their judgement with a ‘seed parameter’ (parameter whose true values are known or can be found within the duration of a study). Calibration scores represent the probability that any differences between expert’s probabilities and observed values of ‘seed parameters’ might have arisen by chance. Information represents the degree to which an expert’s distribution is concentrated, relative to some user-selected background measure | None used |  |  |
| Ethical issues | The ethical issues for the expert sample and research community should be described | The use of expert elicitation should acknowledge the issues of ethical responsibility, anonymity, reliability and validity in an ongoing manner throughout the data collection and aggregation process | Methods, Data collection and Analysing expert values |  |  |
| Presentation of results | The individual, and aggregated, point estimate(s) and distribution for each uncertain quantity (quantities) should be presented | The units of measurement should be clear and attention should be paid to the style of presentation that may benefit from the use of figures rather than relying on a tabular format | Table 1 and Figures 2-4 |  |  |
| Interpretation of results | The interpretation of uncertain quantities elicited should be presented together with a description of how the results will be used in the model-based economic analysis | This should include an explanation of how the reader should interpret the results. It should be recognised that the number and type of experts used will affect the results obtained. The interpretation of results should comment on the degree of uncertainty observed | Results, Expert elicitation of proportions and Unit costs and summary costs |  |  |

### Appendix 2- Options for the diagnosis, primary management and prescription of systemic anti-cancer therapies for melanoma

*Note – where Q in these diagrams refer to the relevant questions in Table 1 in the main text*

|  | Person presents in primary care (or specialist follow-up) with pigmented skin lesion | | | | | | | | | |  |  |  |  |
| --- | --- | --- | --- | --- | --- | --- | --- | --- | --- | --- | --- | --- | --- | --- |
|  |  |  |  |  |  |  |  |  |  |  |  |  |  |  |
|  | Referral to dermatology if clinically suspicious lesion | | | | | | | | | |  |  |  |  |
|  |  |  |  |  |  |  |  |  |  |  |  |  |  |  |
|  | Visual assessment (may include dermoscopy) and palpation of regional lymph nodes | | | | | | | | | |  |  |  |  |
|  |  |  |  |  |  |  |  |  |  |  |  |  |  |  |
|  |  | Q1 |  |  |  |  |  |  |  |  |  |  |  |  |
|  | Clinical melanoma with palpable nodes | | | |  |  | No palpable nodes | | | |  |  |  |  |
|  |  |  |  |  |  |  |  |  |  |  |  |  |  |  |
|  |  |  |  |  | Q2 |  |  |  |  |  |  |  |  |  |
|  |  |  |  | Biopsy of suspicious pigmented lesion | | | |  |  | Clinically not melanoma | | | |  |
|  |  |  |  |  |  |  |  |  |  |  |  |  |  |  |
|  |  | Q3 |  |  |  |  |  |  |  |  |  |  |  |  |
|  | Excision biopsy | | | |  |  | Other (including shave or punch) | | | |  |  |  |  |
|  |  |  |  |  |  |  |  |  |  |  |  |  |  |  |
|  |  |  |  |  |  |  |  |  |  |  |  |  |  |  |
|  |  |  |  | Histopathological report | | | |  |  |  |  |  |  |  |
|  |  |  |  |  |  |  |  |  |  |  |  |  |  |  |
|  |  | Q4 |  |  |  |  |  |  |  |  |  |  |  |  |
|  | Histological diagnosis of melanoma | | | |  |  | Histologically not melanoma | | | |  |  |  |  |
|  |  |  |  |  |  |  |  |  |  |  |  |  |  |  |
|  |  |  |  |  |  |  |  |  |  |  |  |  |  |  |
|  |  | Q5 |  |  |  |  |  |  |  |  |  |  |  |  |
|  | Referral to specialist nurse and surgery | | | |  |  | Direct referral for wide local excision | | | |  |  |  |  |

|  |  |  |  |  |  |  |  |  |  |  |  |  |  |  |  |  |  |  |  |  |  |  |  |  |
| --- | --- | --- | --- | --- | --- | --- | --- | --- | --- | --- | --- | --- | --- | --- | --- | --- | --- | --- | --- | --- | --- | --- | --- | --- |
|  |  | Stage 1b and 2 | | | |  |  |  |  |  | Stage 3 | | | |  |  |  |  | Unresectable stage 3 and stage 4 | | |  |  |  |
|  |  |  |  |  |  |  |  |  |  |  |  |  |  |  |  |  |  |  |  |  |  |  |  |  |
|  | Q1 |  |  |  |  |  |  |  |  |  |  |  |  |  |  |  |  |  |  |  |  |  |  |  |
|  | Imaging (head MRI/PET CT/CT TAP) | | | |  |  |  |  |  |  | Imaging (head MRI/PET CT/CT TAP) | | | | Q6: Upstage to 4 | |  |  |  |  |  |  |  |  |
|  |  |  |  |  |  | Wide local excision | |  |  |  |  |  |  |  |  |  |  |  | SACT | | Q9 | Excision of solitary metastasis | |  |
|  |  |  |  |  |  |  |  |  |  |  |  |  |  |  |  |  |  |  |  |  |  |  |  |  |
|  |  |  |  |  |  |  |  |  |  |  |  |  |  |  |  |  |  |  |  |  |  |  |  |  |
|  |  |  |  |  |  |  |  |  |  |  |  |  |  |  |  |  |  |  |  |  |  |  |  |  |
|  |  |  |  |  |  |  |  |  |  |  |  |  |  |  |  |  |  |  |  |  |  |  |  |  |
|  | Q2 |  |  | Q3 |  |  |  |  |  |  |  |  |  |  |  |  |  |  |  |  |  |  |  |  |
|  | Direct closure | |  | Split/full thickness skin graft | |  | Regional flap | |  |  |  |  |  |  |  |  |  |  |  |  |  |  |  |  |
|  |  |  |  |  |  |  |  |  |  |  |  |  |  |  |  |  |  |  |  |  |  |  |  |  |
|  |  |  |  |  |  |  |  |  |  |  |  |  |  |  |  |  |  |  |  |  |  |  |  |  |
|  |  |  |  |  |  |  |  |  |  |  |  |  |  |  |  |  |  |  |  |  |  |  |  |  |
|  |  |  |  |  |  |  |  |  |  |  |  |  |  |  |  |  |  |  |  |  |  |  |  |  |
|  |  |  | Sentinel lymph node biopsy (stage 1b and 2) | | | Q4 Upstage to 3 | | |  |  |  |  |  |  |  |  |  |  |  |  |  |  |  |  |
|  |  |  |  |  |  |  |  |  |  |  | Q7 |  |  |  |  |  |  |  |  |  |  |  |  |  |
|  |  |  |  |  |  |  |  |  |  | Lymph node dissection | | |  | Adjuvant SACT | |  |  |  |  |  |  |  |  |  |
|  |  |  | Follow-up visits | | |  |  |  |  |  |  |  |  |  |  |  |  |  |  |  |  |  |  |  |
|  |  |  |  |  |  |  |  |  |  |  |  |  |  |  |  |  |  |  |  |  |  |  |  |  |
|  |  |  |  |  |  | Q5 Upstage to 3 | | |  | Follow-up visits | | |  |  |  |  |  |  |  |  |  |  |  |  |
|  |  |  |  |  |  |  |  |  |  |  |  |  |  |  |  |  |  |  |  |  |  |  |  |  |
|  |  |  |  |  |  |  |  |  |  |  |  |  | Q8: Upstage to 4 | | |  |  |  |  |  |  |  |  |  |
|  |  |  |  |  |  |  |  |  |  |  |  |  |  |  |  |  |  |  |  |  |  |  |  |  |

|  |  |  |  |  |  |  |  |  |  |  |  |  |  |  |  |  |  |  |  |  |  |  |  |  |  |  |  |  |  |  |  |  |  |  |  |  |  |  |  |  |
| --- | --- | --- | --- | --- | --- | --- | --- | --- | --- | --- | --- | --- | --- | --- | --- | --- | --- | --- | --- | --- | --- | --- | --- | --- | --- | --- | --- | --- | --- | --- | --- | --- | --- | --- | --- | --- | --- | --- | --- | --- |
|  |  |  |  |  |  |  |  |  |  |  |  |  |  |  |  |  |  |  |  |  |  |  | Stage 4* | | | |  |  |  |  |  |  |  |  |  |  |  |  |  |  |
|  |  |  |  |  |  |  |  |  |  |  |  |  |  |  |  |  |  |  |  |  |  |  |  | Q5 |  |  |  |  |  |  |  |  |  |  |  |  |  |  |  |  |
|  |  |  |  |  |  |  |  |  |  |  |  |  |  |  |  |  |  |  |  |  |  | Genetic testing - BRAF V600 | | | | | |  |  |  |  |  |  |  |  |  |  |  |  |  |
|  |  |  |  |  |  |  |  |  |  |  |  |  |  |  |  |  |  |  |  |  |  |  |  |  |  |  |  |  |  |  |  |  |  |  |  |  |  |  |  |  |
|  |  |  |  |  |  |  |  |  |  |  |  |  |  |  |  |  |  |  | -ve |  |  |  |  |  |  |  |  |  |  | +ve |  |  |  |  |  |  |  |  |  |  |
|  |  |  |  |  |  |  |  |  |  |  |  |  |  |  |  | Q6 |  |  |  |  |  |  |  |  |  |  | Q7 |  |  |  |  |  |  |  |  |  |  |  |  |  |
|  |  |  |  |  |  |  |  |  |  |  |  |  |  |  | Single agent (nivolumab or pembrolizumab) | | | |  | Nivolumab + ipilimumab | | |  | Targeted therapy (encorafenib + binimetinib  or dabrafenib +trametinib) | | | | | | |  |  | Q8 |  |  |  |  |  |  |  |
|  |  |  |  |  |  |  |  |  |  |  |  |  |  |  |  |  |  |  |  |  |  |  |  |  |  |  |  |  |  |  |  | Single agent (nivolumab or pembrolizumab) | | | |  | Nivolumab + ipilimumab | | |  |
|  |  |  |  |  |  |  |  |  |  |  |  |  |  |  |  |  |  |  |  |  |  |  |  |  |  |  |  |  |  |  |  |  |  |  |  |  |  |  |  |  |
|  |  |  |  |  |  | Stage 3 | | | |  |  |  |  |  |  |  |  |  |  |  |  |  |  |  |  |  | Q10 |  |  |  |  |  |  |  |  |  |  |  |  |  |
|  |  |  |  |  |  |  | Q1 |  |  |  |  |  |  |  |  |  |  |  | Q9 |  |  |  |  | Second line immunotherapy | | | | | | |  |  |  |  |  |  |  |  |  |  |
|  |  |  |  |  | Genetic testing - BRAF V600 | | | | | |  |  |  |  |  |  | Ipilimumab | | | | |  |  |  |  |  |  |  |  |  |  |  |  |  | Q12 |  |  |  |  |  |
|  | No adjuvant therapy | | |  |  |  | Q2 |  |  |  |  |  |  |  |  |  |  |  |  |  |  |  |  | Q11 |  |  |  |  |  |  |  |  | Targeted therapy (encorafenib + binimetinib  or dabrafenib +trametinib) | | | | | |  |  |
|  |  |  |  |  |  |  |  |  |  |  |  |  |  |  |  |  |  |  |  |  |  |  | Single agent (nivolumab or pembrolizumab) | | | |  | Nivolumab + ipilimumab | | |  |  |  |  |  |  |  |  |  |  |
|  |  |  |  | |  |  |  |  |  |  |  |  |  |  |  |  |  |  |  |  |  |  |  |  |  |  |  |  |  |  |  |  |  |  |  |  |  |  |  |  |
|  |  |  | Q3 | -ve |  |  |  |  |  | +ve |  |  |  |  |  |  |  |  |  |  |  |  |  |  |  |  |  |  |  |  |  |  |  |  |  |  |  |  |  |  |
|  |  | Single agent (nivolumab or pembrolizumab) | | | |  |  | Q4 |  |  |  |  |  |  |  |  |  |  |  |  |  |  |  |  |  |  |  |  |  |  |  |  | Q13 |  |  |  |  |  |  |  |
|  |  |  |  |  |  |  | Single agent (nivolumab or pembrolizumab) | | | |  | Dabrafenib + trametinib | | |  |  |  |  |  |  |  |  |  |  |  |  |  |  |  |  | *Palliative care can be received at any point in pathway | | | | | | |  |  |  |
|  |  |  |  |  |  |  |  |  |  |  |  |  |  |  |  |  |  |  |  |  |  |  |  |  |  |  |  |  |  |  |  |  |  |  |  |  |  |  |  |  |
|  |  |  |  |  |  |  |  |  |  |  |  |  |  |  |  |  |  |  |  |  |  |  |  |  |  |  |  |  |  |  |  |  |  |  |  |  |  |  |  |  |
|  |  |  |  |  |  |  |  |  |  |  |  |  |  |  |  |  |  |  |  |  |  |  |  |  |  |  |  |  |  |  |  |  |  |  |  |  |  |  |  |  |

### Appendix 3 – Information used to calculate total costs

**Table A3.1 – Proportions not elicited from experts**

| **Proportion** | **Value** | **Source** | **Further details** |
| --- | --- | --- | --- |
| Proportion of stage 3 melanoma cases which are BRAF positive | 34% | National Institute for Health and Care Excellence (2015)^1^ | [Evidence review A](https://www.nice.org.uk/guidance/ng14/evidence/health-economic-model-report-for-evidence-review-a-pdf-11186298254)^2^, table HE013 |
| Proportion of stage 4 melanoma cases which are BRAF positive | 34% | National Institute for Health and Care Excellence (2015)^1^ | [Evidence review A](https://www.nice.org.uk/guidance/ng14/evidence/health-economic-model-report-for-evidence-review-a-pdf-11186298254)^2^, table HE013 |
| Proportion of imaging which is chest/abdomen/pelvis | 50% | National Institute for Health and Care Excellence (2015)^1^ | [Committee papers](https://www.nice.org.uk/guidance/ta837/documents/committee-papers)^3^, table 50, p. 105 |
| Proportions of single agent systemic therapy which is nivolumab | 50% | Assumption |  |
| Proportion of targeted treatment which is encorafenib + binimetanib | 50% | Assumption |  |
| Proportion of SLNB mapping to neck procedures | 14% | Rughani, M.G. *et al.* (2011)^4^ | p. 3, sum of neck, parotid and bilateral neck |
| Proportion of SLNB mapping to unilateral breast procedure | 44% | Rughani, M.G. *et al.* (2011)^4^ | p. 3, axillary lymph node |
| Proportion of SLNB happing to abdomen procedures | 37% | Rughani, M.G. *et al.* (2011)^4^ | p. 3, sum of groin and bilateral groin |
| Proportion of SLNB mapping to bilateral breast | 4% | Rughani, M.G. *et al.* (2011)^4^ | p. 3, bilateral axillae |
| Proportion of SLNB mapping to procedures on rest of lymphatic system | 1% | Rughani, M.G. *et al.* (2011)^4^ | p. 3, sum of popliteal fossa and epitrochlear |

**Table A3.2 – unit costs**

| **Unit costs (£; 2024/25)** | **Value** | **Source** | **Further details** |
| --- | --- | --- | --- |
| Multidisciplinary team nurse (per hour of time)^a^ | £57 | Jones, K.C. *et al.* (2022)^5^ | Band 6 nurse |
| Imaging of chest/abdomen/pelvis | £126.6 | NHS England (2025)^6^ | Weighted average of the following HRGs: RD20A, RD21A and RD22Z |
| Brain imaging | £197.5 | NHS England (2025)^6^ | Weighted average of the following HRGs: RD01A, RD02A, RD03Z |
| Simple chemotherapy administration | £406 | NHS England (2025)^6^ | HRG=SB12Z |
| Complex chemotherapy administration | £521 | NHS England (2025)^6^ | HRG=SB13Z |
| encorafenib per month^b^ | £6,087.48 | National Institute for Health and Care Excellence (2015)^1^ | [Evidence review F](https://www.nice.org.uk/guidance/ng14/update/ng14-update-1/documents/economic-report-2)^7^, Table HE010 |
| binimetanib per month^b^ | £4,870 | National Institute for Health and Care Excellence (2015)^1^ | [Evidence review F](https://www.nice.org.uk/guidance/ng14/update/ng14-update-1/documents/economic-report-2)^7^, Table HE010 |
| dabrafenib per month^b^ | £6,087.48 | National Institute for Health and Care Excellence (2015)^1^ | [Evidence review F](https://www.nice.org.uk/guidance/ng14/update/ng14-update-1/documents/economic-report-2)^7^, Table HE010 |
| trametinib per month^b^ | £4,870 | National Institute for Health and Care Excellence (2015)^1^ | [Evidence review F](https://www.nice.org.uk/guidance/ng14/update/ng14-update-1/documents/economic-report-2)^7^, Table HE010 |
| nivolumab per month^b^ | £5,705 | National Institute for Health and Care Excellence (2015)^1^ | [Evidence review F](https://www.nice.org.uk/guidance/ng14/update/ng14-update-1/documents/economic-report-2)^7^, Table HE010 |
| pembrolizumab/month^b^ | £7,598 | National Institute for Health and Care Excellence (2015)^1^ | [Evidence review F](https://www.nice.org.uk/guidance/ng14/update/ng14-update-1/documents/economic-report-2)^7^, Table HE010 |
| nivolumab per month (with ipilimumab) ^b^ | £1,268 | National Institute for Health and Care Excellence (2015)^1^ | [Evidence review F](https://www.nice.org.uk/guidance/ng14/update/ng14-update-1/documents/economic-report-2)^7^, Table HE010 |
| ipilimumab per month^b^ | £27,083 | National Institute for Health and Care Excellence (2015)^1^ | [Evidence review F](https://www.nice.org.uk/guidance/ng14/update/ng14-update-1/documents/economic-report-2)^7^, Table HE010 |
| SLNB of neck, parotid or bilateral neck | £2,631 | NHS England (2025)^6^ | HRG=CA05A, daycase |
| SLNB of axillary lymph node | £2,715 | NHS England (2025)^6^ | HRG=JA43B, daycase |
| SLNB of groin or bilateral groin | £3,353 | NHS England (2025)^6^ | HRG=FF51e, daycase |
| SLNB of bilateral axillae | £3,630 | NHS England (2025)^6^ | HRG=JA42Z, daycase |
| SLNB of popliteal fossa or epitrochlear | £1,644 | NHS England (2025)^6^ | HRG=WH54B, daycase |

**Table A3.3 – resource use**

| **Resource use** | **Value** | **Source** | **Further Detail** |
| --- | --- | --- | --- |
| Number of appointments for S1b-2, not upstaged | 9 | National Institute for Health and Care Excellence (2015)^1^ | [Recommendations](https://www.nice.org.uk/guidance/ng14/chapter/recommendations), unweighted average of follow-up for each stage |
| Number of appointments for S1b-2, upstaged | 4.5 | [Assumption](https://www.nice.org.uk/guidance/ng14/chapter/Recommendations#follow-up-after-treatment-for-melanoma) | Half full follow-up |
| Number of appointments for S3,  not upstaged | 16 | National Institute for Health and Care Excellence (2015)^1^ | [Recommendations](https://www.nice.org.uk/guidance/ng14/chapter/recommendations), 4 appts. in years 1-3, 2 appts. in yrs. 4 and 5 |
| Number of appointments for S3, upstaged | 8 | [Assumption](https://www.nice.org.uk/guidance/ng14/chapter/Recommendations#follow-up-after-treatment-for-melanoma) | Half full follow-up |
| Number of appointments for S4 | 6 | Assumption |  |
| Appointment with multi-disciplinary team nurse (minutes) | 60 minutes | Assumption |  |
| Time on treatment for pembrolizumab (months) | 6.18 months | National Institute for Health and Care Excellence (2015)^1^ | [Evidence review F](https://www.nice.org.uk/guidance/ng14/update/ng14-update-1/documents/economic-report-2)^7^, p. 17, median |
| Time on treatment for nivolumab (months) | 8.64 months | National Institute for Health and Care Excellence (2015)^1^ | [Evidence review F](https://www.nice.org.uk/guidance/ng14/update/ng14-update-1/documents/economic-report-2)^7^, p. 19, median |
| Time on treatment for nivolumab + ipilumumab (months) | 2.04 months | National Institute for Health and Care Excellence (2015)^1^ | [Evidence review F](https://www.nice.org.uk/guidance/ng14/update/ng14-update-1/documents/economic-report-2)^7^, p. 21, median |
| Time on treatment for trametinib + dabrafenib (months) | 15.7 months | National Institute for Health and Care Excellence (2015)^1^ | Evidence review F^7^, p. 24 |
| Time on treatment for encorafenib + binimetinib (months) | 16.9 months | National Institute for Health and Care Excellence (2015)^1^ | Evidence review F^7^, p. 24 |
| Time on treatment for ipilimumab (months) | 7.77 months | National Institute for Health and Care Excellence (2015)^1^ | Evidence review F^7^, 2nd line ToT after single nivolumab/pembrolizumab (p. 38) |

**Table A3.4 – service costs**

| **Service costs (£; 2024/25)** | **Value** | **Source** | **Further Detail** |
| --- | --- | --- | --- |
| *Diagnosis* |  |  |  |
| General Practitioner Appointment ^a^ | £45 | Jones, K.C. *et al.* (2025)^5^ | Including direct care and qualifications |
| Dermatology consultation | £165 | NHS England (2025)^6^ | HRG=330, consultant lead |
| Dermoscopy | £215 | NHS England (2025)^6^ | HRG=JC43C , outpatient |
| Excision biopsy | £215 | NHS England (2025)^6^ | HRG=JC43C, outpatient |
| Shave or punch biopsy | £215 | NHS England (2025)^6^ | HRG=JC43C, outpatient |
| Histopathology | £60.05 | NHS England (2025)^6^ | HRG=PATH02 |
| *Primary Management* |  |  |  |
| Multidisciplinary team referral | £57 | Calculation | 1 hr. multidisciplinary nurse time |
| WLE direct closure | £215 | NHS England (2025)^6^ | Outpatient, HRG=JC43C, mapping based on^8^ https://cdn.bad.org.uk/uploads/2023/02/02135037/Coding-Booklet-2023-updated-31.01.2023.pdf |
| WLE graft repair | £264 | NHS England (2025)^6^ | Outpatient, HRG=JC42C, mapping based on^8^ https://cdn.bad.org.uk/uploads/2023/02/02135037/Coding-Booklet-2023-updated-31.01.2023.pdf |
| WLE flap repair | £1650 | NHS England (2025)^6^ | Daycase, HRG=JC41Z, mapping based on^8^ https://cdn.bad.org.uk/uploads/2023/02/02135037/Coding-Booklet-2023-updated-31.01.2023.pdf |
| Imaging | £360 | Calculation | Brain MRI cost + 50:50 average of PET/CT (as per NICE TA837, cost of MRI used as proxy in absence of HRG for PET/CT) and contrast CT for chest/abdomen/pelvis |
| Lymph node dissection | £1644 | NHS England (2025)^6^ | HRG=WH54B, daycase |
| *Systemic Anti-Cancer Therapies* |  |  |  |
| Genetic test^b^ | £75 | National Institute for Health and Care Excellence (2015)^1^ | [Evidence review A](https://www.nice.org.uk/guidance/ng14/evidence/health-economic-model-report-for-evidence-review-a-pdf-11186298254)^2^, Table HE1.3.7.1, p. 18 |
| nivolumab course | £49,810.49 | Calculation | Monthly cost multiplied by estimated months on treatment + adminstration HRG |
| pembrolizumab course | £47,360.56 | Calculation | Monthly cost multiplied by estimated months on treatment + adminstration HRG |
| nivolumab + ipilimumab course | £57,837.16 | Calculation | Monthly cost multiplied by estimated months on treatment |
| dabrafenib + trametinib course | £172,032.44 | Calculation | Monthly cost multiplied by estimated months on treatment |
| Targeted Therapy course (encorafenib + binimetinib/ dabrafenib +trametinib) | £178,606.92 | Calculation | Monthly cost multiplied by estimated months on treatment |
| Ipilimumab course | £210,437.47 | Calculation | Monthly cost multiplied by estimated months on treatment |
| CT=computed tomography, HRG=healthcare resource group, PET=positron emission topography, SLNB=sentinel lymph node biopsy, WLE=wide local excision | | | |

Footnotes:
a. 2023/2024 costs, which reflect the most current figures.
b. 2022 costs.

**References**

1. National Institute for Health and Care Excellence. Overview | Melanoma: assessment and management | Guidance | NICE (updated 2022), https://www.nice.org.uk/guidance/ng14 (2015, accessed 23 December 2025).

2. *Evidence reviews for genetic testing for melanoma: Melanoma: assessment and management: Evidence review A*. London: National Institute for Health and Care Excellence (NICE), http://www.ncbi.nlm.nih.gov/books/NBK588625/ (2022, accessed 23 December 2025).

3. National Institute for Health and Care Excellence. *Pembrolizumab for adjuvant treatment of resected stage 2 melanoma with high risk of recurrence: committee papers*, https://www.nice.org.uk/guidance/ta837/documents/committee-papers (2022).

4. Rughani MG, Swan MC, Adams TS, et al. Sentinel lymph node biopsy in melanoma: The Oxford ten year clinical experience. *J Plast Reconstr Aesthet Surg* 2011; 64: 1284–1290.

5. Jones KC, Weatherly H, Birch S, et al. Unit Costs of Health and Social Care 2024 Manual. Epub ahead of print 8 April 2025. DOI: 10.22024/UniKent/01.02.109563.

6. NHS England. National Cost Collection for the NHS, https://www.england.nhs.uk/costing-in-the-nhs/national-cost-collection/ (accessed 19 December 2025).

7. National Institute for Health and Care Excellence. *Evidence reviews for systemic and localised anticancer treatment for people with stage IV and unresectable stage III melanoma: Melanoma: assessment and management: Evidence review F*. London: National Institute for Health and Care Excellence (NICE), http://www.ncbi.nlm.nih.gov/books/NBK588629/ (2022, accessed 23 December 2025).

8. British Association of Dermatologists. *The Science of Casemix: Under the Microscope: Dermatology coding for non-specialised services*, https://cdn.bad.org.uk/uploads/2023/02/02135037/Coding-Booklet-2023-updated-31.01.2023.pdf (2020).

### Appendix 4- Results of the sensitivity analysis based on linear pooling method for combining experts’ estimates

| **Item** | **Unit cost**  **(£; 2024/25)** | **Expected mean cost – £ (95% confidence interval)** | | | | | | | |
| --- | --- | --- | --- | --- | --- | --- | --- | --- | --- |
|  |  | **Lesion not melanoma** | | **Stage 0** | **Stage 1a** | **Stage 1b/2** | **Stage 3** | | **Stage 4** |
|  |  | **Diagnosed in clinic** | **Requiring biopsy** |  |  |  | **Microscopic** | **Macroscopic** |  |
| **Diagnosis** |  |  |  |  |  |  |  |  |  |
| GP appointment | 45 | 45 | 45 | 45 | 45 | 45 | – | 45 | – |
| Dermatology appointment | 165 | 165 | 165 | 165 | 165 | 165 | – | 165 | – |
| Dermoscopy | 215 | 215 | 215 | 215 | 215 | 215 | – | – | – |
| Excision Biopsy | 215 | – | 194 (152–213) | 194 (152–213) | 194 (152–213) | 194 (152–213) | – | 215 | – |
| Shave/punch biopsy | 215 | – | 21 (2–62) | 21 (2–62) | 21 (2–62) | 21 (2–62) | – | – | – |
| Histopathology | 60 | – | 60 | 60 | 60 | 60 | - | 60 | – |
| Diagnosis total |  | 424 | 699 (699–699)* | 699 (699–699)* | 699 (699–699)* | 699 (699–699)* | 0 (0–0) | 484 | 0 (0–0) |
| **Management** |  |  |  |  |  |  |  |  |  |
| Specialist nurse | – | – | 53 (48–57) | 53 (48–57) | 53 (48–57) | 53 (48–57) | – | – | – |
| Wide local excision direct closure | – | – | 145 (67–202) | 145 (67–202) | 145 (67–202) | – | – | – | – |
| Wide local excision graft | – | – | 21 (1–72) | 21 (1–72) | 21 (1–72) | – | – | – | – |
| Wide local excision flap | – | – | 399 (65–921) | 399 (65–921) | 399 (65–921) | – | – | – | – |
| 1x follow-up after Wide local excision | – | – | 165 | – | – | – | – | – | – |
| 2x follow up after Wide local excision | – | – | – | 329 | – | – | – | – | – |
| Sentinel lymph node biopsy | – | – | – | – | 2970 | – | – | – | – |
| Imaging (brain MRI + PETCT or CT of thorax/abdomen/pelvis) | – | – | – | – | 56 (2–170) | 360 | – | 360 | – |
| Lymph node dissection | – | – | – | – | – | 1644 | – | 91 (20–211) | – |
| Histopathology |  |  |  |  |  | 60 |  | 3 (1–8) |  |
| Complete 5-year follow-up (stage 1b/2) | – | – | – | – | 1,008 (835–1,156) | – | – | – | – |
| Upstage during 5-year follow-up (stage 1b/2) | – | – | – | – | 85 (29–166) | – | – | – | – |
| Complete 5-year follow-up (stage 3) | – | – | – | – | – | 1,372 (757–1,913) | – | 2,071 (1,737–2,346) | – |
| Upstage during 5-year follow-up (stage 3) | – | – | – | – | – | 186 (77–335) | – | 281 (143–448) | – |
| Management total |  | 0 (0–0) | 0 (0–0) | 784 (490–1,239) | 948 (655–1,403) | 4,738 (4,400–5,211) | 2,806 (2,617–2,992) | 3,676 (2,988–4,241) | 0 (0–0) |
| **Systemic anti-cancer therapies (SACT)** |  |  |  |  |  |  |  |  |  |
| Genetic test (BRAF) | 75 | – | – | – | – | – | 58 (47–67) | 58 (47–67) | 72 (65–75) |
| Single agent nivolumab/pembrolizumab | 48,586 | – | – | – | – | – | 26,187 (16,374–37,962) | 26,187 (16,374–37,962) | 20,658 (9,997–32,290) |
| Nivolumab + ipilumimab | 57,837 | – | – | – | – | – | 4,475 (272–12,937) | 4,475 (272–12,937) | 30,590 (16,701–43,332) |
| Dabrafenib + trametinib | 172,032 | – | – | – | – | – | 31,649 (996–49,944) | 31,649 (996–49,944) | – |
| Targeted therapy (dabrafenib +trametinib or encorafenib +binimetinib) | 178,607 | – | – | – | – | – | – | – | 8,200 (2,752–15,718) |
| 6 × follow-up (stage 4 only) | 987 | – | – | – | – | – | – | – | 987 |
| Single agent nivolumab/pembrolizumab (2^nd^ line) | 48,586 | – | – | – | – | – | – | – | 549 (34–1,774) |
| Nivolumab+ipilimumab (2^nd^ line) | 57,837 | – | – | – | – | – | – | – | 320 (11–1,213) |
| Ipilimumab (2^nd^ line) | 210,437 | – | – | – | – | – | – | – | 43,676 (8,259–93,676) |
| Targeted therapy (dabrafenib+trametinib or encorafenib+binimetinib) (2^nd^ line) | 178,607 | – | – | – | – | – | – | – | 30,524 (5,574–50,727) |
| 6x follow-up (stage 4) | 987 | – | – | – | – | – | – | – | 987 |
| SACT total |  | 0 (0–0) | 0 (0–0) | 0 (0–0) | 0 (0–0) | 0 (0–0) | 62,370 (38,892–80,010) | 62,370 (38,892–80,010) | 135,570 (90,337–189,154) |

*no variation as the two alternative diagnostic options have the same unit cost

### Appendix 5- Sensitivity analysis showing total costs calculated using responses for each individual expert

| **Specialty** | **Expert** | **Category** | **Expected cost (£; 2024/25)** | | | | | | | |
| --- | --- | --- | --- | --- | --- | --- | --- | --- | --- | --- |
|  |  |  | **Lesion not melanoma** | | **Stage 0** | **Stage 1a** | **Stage 1b/2** | **Stage 3** | | **Stage 4** |
|  |  |  | **Diagnosed in clinic** | **Requiring biopsy** |  |  |  | **Microscopic** | **Macroscopic** |  |
| Dermatology | 1 | Diagnosis | 424 | 699 | 699 | 699 | 699 | – | 484 | – |
|  |  | Primary management | – | – | 693 | 857 | 4,688 | 2,811 | 4,066 | – |
|  |  | SACT | – | – | – | – | – | 55,012 | 55,012 | 132,802 |
|  | 2 | Diagnosis | 424 | 699 | 699 | 699 | 699 | – | 484 | – |
|  |  | Primary management | – | – | 675 | 840 | 4,595 | 2,796 | 4,074 | – |
|  |  | SACT | – | – | – | – | – | 66,507 | 66,507 | 120,947 |
|  | 3 | Diagnosis | 424 | 699 | 699 | 699 | 699 | – | 484 | – |
|  |  | Primary management | – | – | 870 | 1,034 | 4,759 | 2,802 | 3,917 | – |
|  |  | SACT | – | – | – | – | – | 62,477 | 62,477 | 137,328 |
|  | 4 | Diagnosis | 424 | 699 | 699 | 699 | 699 | – | 484 | – |
|  |  | Primary management | – | – | 887 | 1,051 | 4,807 | 2,791 | 3,790 | – |
|  |  | SACT | – | – | – | – | – | 67,941 | 67,941 | 134,519 |
|  | 5 | Diagnosis | 424 | 699 | 699 | 699 | 699 | – | 484 | – |
|  |  | Primary management | – | – | 951 | 1,116 | 4,901 | 2,800 | 3,325 | – |
|  |  | SACT | – | – | – | – | – | 66,074 | 66,074 | 141,542 |
|  | 6 | Diagnosis | 424 | 699 | 699 | 699 | 699 | – | 484 | – |
|  |  | Primary management | – | – | 824 | 988 | 4,752 | 2,821 | 3,561 | – |
|  |  | SACT | – | – | – | – | – | 70,124 | 70,124 | 137,786 |
|  | 7 | Diagnosis | 424 | 699 | 699 | 699 | 699 | – | 484 | – |
|  |  | Primary management | – | – | 793 | 957 | 4,683 | 2,797 | 3,883 | – |
|  |  | SACT | – | – | – | – | – | 65,425 | 65,425 | 136,065 |
| Surgery | 1 | Diagnosis | 424 | 699 | 699 | 699 | 699 | – | 484 | – |
|  |  | Primary management | – | – | 723 | 887 | 4,642 | 2,841 | 3,659 | – |
|  |  | SACT | – | – | – | – | – | 72,178 | 72,178 | 146,773 |
|  | 2 | Diagnosis | 424 | 699 | 699 | 699 | 699 | – | 484 | – |
|  |  | Primary management | – | – | 1,026 | 1,191 | 5,068 | 2,709 | 3,679 | – |
|  |  | SACT | – | – | – | – | – | 63,793 | 63,793 | 131,578 |
|  | 3 | Diagnosis | 424 | 699 | 699 | 699 | 699 | – | 484 | – |
|  |  | Primary management | – | – | 679 | 843 | 4,586 | 2,804 | 3,278 | – |
|  |  | SACT | – | – | – | – | – | 63,313 | 63,313 | 140,500 |
|  | 4 | Diagnosis | 424 | 699 | 699 | 699 | 699 | – | 484 | – |
|  |  | Primary management | – | – | 680 | 845 | 4,621 | 2,832 | 4,129 | – |
|  |  | SACT | – | – | – | – | – | 52,110 | 52,110 | 135,468 |
| Oncology | 1 | Diagnosis | 424 | 699 | 699 | 699 | 699 | – | 484 | – |
|  |  | Primary management | – | – | 743 | 908 | 4,594 | 2,826 | 3,602 | – |
|  |  | SACT | – | – | – | – | – | 57,269 | 57,269 | 165,181 |
|  | 2 | Diagnosis | 424 | 699 | 699 | 699 | 699 | – | 484 | – |
|  |  | Primary management | – | – | 785 | 949 | 4,752 | 2,888 | 4,020 | – |
|  |  | SACT | – | – | – | – | – | 49,690 | 49,690 | 94,117 |
|  | 3 | Diagnosis | 424 | 699 | 699 | 699 | 699 | – | 484 | – |
|  |  | Primary management | – | – | 666 | 831 | 4,652 | 2,841 | 3,367 | – |
|  |  | SACT | – | – | – | – | – | 75,946 | 75,946 | 132,493 |
|  | 4 | Diagnosis | 424 | 699 | 699 | 699 | 699 | – | 484 | – |
|  |  | Primary management | – | – | 870 | 1,034 | 4,888 | 2,837 | 4,019 | – |
|  |  | SACT | – | – | – | – | – | 64,371 | 64,371 | 155,158 |
|  | 5 | Diagnosis | 424 | 699 | 699 | 699 | 699 | – | 484 | – |
|  |  | Primary management | – | – | 839 | 1,004 | 4,816 | 2,754 | 3,812 | – |
|  |  | SACT | – | – | – | – | – | 66,676 | 66,676 | 130,770 |

### Appendix 6 - Sensitivity analysis showing total costs calculated using hypothetical values for the unit costs of systemic anti-cancer therapies (SACT)

**Table A6.1– SACT costs reduced to 20% of list price**

| **Item** | **Unit cost**  **(£; 2024/25)** | **Expected mean cost – £ (95% confidence interval)** | | | | | | | |
| --- | --- | --- | --- | --- | --- | --- | --- | --- | --- |
|  |  | **Lesion not melanoma** | | **Stage 0** | **Stage 1a** | **Stage 1b/2** | **Stage 3** | | **Stage 4** |
|  |  | **Diagnosed in clinic** | **Requiring biopsy** |  |  |  | **Microscopic** | **Macroscopic** |  |
| **Systemic anti-cancer therapies (SACT)** |  |  |  |  |  |  |  |  |  |
| Genetic test (BRAF) | 75 | – | – | – | – | – | 58 (54–62) | 58 (54–62) | 71 (69–73) |
| Single agent nivolumab/pembrolizumab | 10,088 | – | – | – | – | – | 5,343 (4,410–6,637) | 5,343 (4,410–6,637) | 4,208 (2,848–5,635) |
| Nivolumab + ipilumimab | 11,567 | – | – | – | – | – | 811 (345–1,463) | 811 (345–1,463) | 6,249 (4,627–7,802) |
| Dabrafenib + trametinib | 34,406 | – | – | – | – | – | 7,097 (2,994–9,248) | 7,097 (2,994–9,248) | – |
| Targeted therapy (dabrafenib +trametinib or encorafenib +binimetinib) | 35,721 | – | – | – | – | – | – | – | 1,523 (1,019–2,099) |
| 6 × follow-up (stage 4 only) | 987 | – | – | – | – | – | – | – | 987 |
| Single agent nivolumab/pembrolizumab (2^nd^ line) | 10,088 | – | – | – | – | – | – | – | 104 (30–212) |
| Nivolumab+ipilimumab (2^nd^ line) | 11,567 | – | – | – | – | – | – | – | 56 (14–128) |
| Ipilimumab (2^nd^ line) | 42,087 | – | – | – | – | – | – | – | 8,228 (5,550–11,214) |
| Targeted therapy (dabrafenib+trametinib or encorafenib+binimetinib) (2^nd^ line) | 35,721 | – | – | – | – | – | – | – | 6,403 (3,969–8,561) |
| 6x follow-up (stage 4) | 987 | – | – | – | – | – | – | – | 987 |
| SACT total |  | 0 (0–0) | 0 (0–0) | 0 (0–0) | 0 (0–0) | 0 (0–0) | 13,309 (10,339–15,194) | 13,309 (10,339–15,194) | 27,830 (24,143–31,497) |

**Table A6.2– SACT costs reduced to 40% of list price**

| **Item** | **Unit cost**  **(£; 2024/25)** | **Expected mean cost – £ (95% confidence interval)** | | | | | | | |
| --- | --- | --- | --- | --- | --- | --- | --- | --- | --- |
|  |  | **Lesion not melanoma** | | **Stage 0** | **Stage 1a** | **Stage 1b/2** | **Stage 3** | | **Stage 4** |
|  |  | **Diagnosed in clinic** | **Requiring biopsy** |  |  |  | **Microscopic** | **Macroscopic** |  |
| **Systemic anti-cancer therapies (SACT)** |  |  |  |  |  |  |  |  |  |
| Genetic test (BRAF) | 75 | – | – | – | – | – | 58 (54–62) | 58 (54–62) | 71 (69–73) |
| Single agent nivolumab/pembrolizumab | 19,712 | – | – | – | – | – | 10,436 (8,554–13,055) | 10,436 (8,554–13,055) | 8,223 (5,597–10,958) |
| Nivolumab + ipilumimab | 23,135 | – | – | – | – | – | 1,616 (684–2,917) | 1,616 (684–2,917) | 12,495 (9,284–15,598) |
| Dabrafenib + trametinib | 68,813 | – | – | – | – | – | 14,203 (5,915–18,453) | 14,203 (5,915–18,453) | – |
| Targeted therapy (dabrafenib +trametinib or encorafenib +binimetinib) | 71,443 | – | – | – | – | – | – | – | 3,052 (2,058–4,189) |
| 6 × follow-up (stage 4 only) | 987 | – | – | – | – | – | – | – | 987 |
| Single agent nivolumab/pembrolizumab (2^nd^ line) | 19,712 | – | – | – | – | – | – | – | 203 (58–416) |
| Nivolumab+ipilimumab (2^nd^ line) | 23,135 | – | – | – | – | – | – | – | 112 (29–265) |
| Ipilimumab (2^nd^ line) | 84,175 | – | – | – | – | – | – | – | 16,518 (11,007–22,461) |
| Targeted therapy (dabrafenib+trametinib or encorafenib+binimetinib) (2^nd^ line) | 71,443 | – | – | – | – | – | – | – | 12,858 (8,051–17,237) |
| 6x follow-up (stage 4) | 987 | – | – | – | – | – | – | – | 987 |
| SACT total |  | 0 (0–0) | 0 (0–0) | 0 (0–0) | 0 (0–0) | 0 (0–0) | 26,314 (20,329–29,981) | 26,314 (20,329–29,981) | 54,519 (47,072–61,910) |

**Table A6.3– SACT costs reduced to 60% of list price**

| **Item** | **Unit cost**  **(£; 2024/25)** | **Expected mean cost – £ (95% confidence interval)** | | | | | | | |
| --- | --- | --- | --- | --- | --- | --- | --- | --- | --- |
|  |  | **Lesion not melanoma** | | **Stage 0** | **Stage 1a** | **Stage 1b/2** | **Stage 3** | | **Stage 4** |
|  |  | **Diagnosed in clinic** | **Requiring biopsy** |  |  |  | **Microscopic** | **Macroscopic** |  |
| **Systemic anti-cancer therapies (SACT)** |  |  |  |  |  |  |  |  |  |
| Genetic test (BRAF) | 75 | – | – | – | – | – | 58 (54–62) | 58 (54–62) | 71 (69–73) |
| Single agent nivolumab/pembrolizumab | 29,337 | – | – | – | – | – | 15,539 (12,788–19,312) | 15,539 (12,788–19,312) | 12,380 (8,379–16,476) |
| Nivolumab + ipilumimab | 34,702 | – | – | – | – | – | 2,428 (994–4,361) | 2,428 (994–4,361) | 18,575 (13,763–23,252) |
| Dabrafenib + trametinib | 103,219 | – | – | – | – | – | 21,279 (9,598–27,765) | 21,279 (9,598–27,765) | – |
| Targeted therapy (dabrafenib +trametinib or encorafenib +binimetinib) | 107,164 | – | – | – | – | – | – | – | 4,583 (3,089–6,298) |
| 6 × follow-up (stage 4 only) | 987 | – | – | – | – | – | – | – | 987 |
| Single agent nivolumab/pembrolizumab (2^nd^ line) | 29,337 | – | – | – | – | – | – | – | 299 (92–610) |
| Nivolumab+ipilimumab (2^nd^ line) | 34,702 | – | – | – | – | – | – | – | 169 (43–381) |
| Ipilimumab (2^nd^ line) | 126,262 | – | – | – | – | – | – | – | 24,826 (16,693–33,675) |
| Targeted therapy (dabrafenib+trametinib or encorafenib+binimetinib) (2^nd^ line) | 107,164 | – | – | – | – | – | – | – | 19,175 (11,878–25,676) |
| 6x follow-up (stage 4) | 987 | – | – | – | – | – | – | – | 987 |
| SACT total |  | 0 (0–0) | 0 (0–0) | 0 (0–0) | 0 (0–0) | 0 (0–0) | 39,304 (30,788–44,960) | 39,304 (30,788–44,960) | 81,062 (70,311–91,894) |
